# Gapless fracton quantum spin liquid and emergent photons in a 2D spin-1 model

**DOI:** 10.1038/s41467-026-74797-0

**Published:** 2026-07-11

**Authors:** Nils Niggemann, Meghadeepa Adhikary, Yannik Schaden-Thillmann, Johannes Reuther

**Affiliations:** 1 Dahlem Center for Complex Quantum Systems and Fachbereich Physik, Freie Universität Berlin, Berlin, Germany; 2 The Abdus Salam International Center for Theoretical Physics (ICTP), Strada Costiera 11, Trieste, Italy; 3 Helmholtz-Zentrum Berlin für Materialien und Energie, Hahn-Meitner Platz 1, Berlin, Germany; 4 Department of Physics and Quantum Centers in Diamond and Emerging Materials (QuCenDiEM) group, Indian Institute of Technology Madras, Chennai, India; 5 SISSA, Via Bonomea 265, Trieste, Italy

**Keywords:** Quantum fluids and solids, Topological matter

## Abstract

Gapless fracton quantum spin liquids are exotic phases of matter described by higher-rank U(1) gauge theories, which host gapped and immobile fracton matter excitations as well as gapless photons. Despite well-known field theories, no spin models beyond purely classical systems have been identified to realize these phases. Using error-controlled Green function Monte Carlo, here we investigate a square lattice spin-1 model that shows precise signatures of a fracton quantum spin liquid without indications of conventional ordering. Specifically, the magnetic response exhibits characteristic patterns of suppressed pinch points that accurately match the prediction of a rank-2 U(1) field theory and reveals the existence of emergent photon excitations in 2+1 spacetime dimensions. Remarkably, this type of fracton quantum spin liquid is not only identified in the system’s ground state but also in generic low-energy sectors of a strongly fragmented Hilbert space.

## Abstract (web summary)

Gapless fracton quantum spin liquids described by higher rank gauge theories have remained elusive in microscopic quantum spin models. Here, the authors demonstrate a spin 1 square lattice model exhibiting signatures of a rank 2 U(1) fracton quantum spin liquid, including suppressed pinch points and emergent photon like excitations revealed by Green function Monte Carlo simulations.

## Introduction

Quantum spin liquids (QSLs) are long-range entangled quantum phases with fractional spin excitations that cannot be smoothly deformed into conventional ordered phases^1,2^. While elusive in nature, their theoretical description follows the established framework of gauge theories, in which different QSLs are characterized by different types of gauge fields^3^. In the most well-studied cases, gauge fields are of ${{\mathbb{Z}}}_{2}$^4^ or U(1) type^5^ and are supplemented by ‘matter’ fields, also referred to as spinons or charges *ρ*. Specifically, the U(1) case offers the remarkable possibility of realizing an emergent quantum electrodynamics (QED) theory in a QSL which manifests in an effective Gauss’ law ***∇*** ⋅ ***E*** = *ρ* for the gauge field ***E*** as well as photon excitations^6–8^. Recently, it has been recognized that U(1) gauge fields allow for an intriguing generalization beyond conventional QED to higher-rank U(1) gauge theories^9–12^, where the gauge fields *E*^*μν*^ are matrices (or higher rank tensors). Specifically, in the rank-2 case and for scalar charges *ρ,* the generalized Gauss’ law reads as ∂_*μ*_∂_*ν*_*E*^*μν*^ = *ρ*. This modification gives rise to unconventional conservation laws, where not only *ρ* but also the dipole moment ***r****ρ* is conserved, which implies that charges *ρ* – in this case called fractons^13–16^ – lose their mobility^17–20^. In so-called type-I fracton theories^17,19,21^, dipoles of charges (named ‘lineons’) still retain a partial mobility along subdimensional manifolds, while in type-II fracton phases^20,22,23^ all composite charges are immobile. In addition to matter particles, a fractonic rank-2 U(1) QSL hosts gauge excitations given by gapless photons, similar to conventional QED. These gapless U(1) fracton systems should be contrasted with their *gapped* cousins, most prominently the X-Cube model^18,24^ and Haah’s code^20^, which exhibit ${{\mathbb{Z}}}_{2}$-valued gauge fields giving rise to *fracton topological order*.

A general obstacle in the gauge theory description of QSLs is the difficulty of establishing a rigorous connection to microscopic interacting spin models. The knowledge of a parent spin Hamiltonian, however, is essential for the search and identification of QSLs in materials or synthetic platforms. Such a connection is only known for very few models. For example, the celebrated Kitaev honeycomb model^25^ exactly realizes a QSL where Majorana fermions couple to ${{\mathbb{Z}}}_{2}$ gauge fields. Equally iconic, quantum spin ice^26,27^ as realized in a spin-1/2 XXZ model on the pyrochlore lattice can be mapped onto a compact U(1) gauge theory and, hence, gives rise to emergent QED^6–8^. In both cases, remarkable experimental progress has been made in recent years to identify these phases in magnetic materials^28–38^. In contrast, higher-rank U(1) gauge theories for fractonic QSLs have so far only been identified in classical spin models on the purely electrostatic level^39–44^. The actual quantum phase of a gapless fracton spin liquid has remained elusive^45^ and its photon mode has completely resisted any description in terms of spin models. The difficulties in capturing fractonic properties in realistic spin systems also exist for gapped fracton models, which typically require rather unrealistic multi-spin interactions^18,20^.

In this paper, we fill this gap by introducing a surprisingly simple spin-1 model on the square lattice that imposes a rank-2 Gauss law constraint, leading to fractionalized excitations (fractons), a hallmark signature of QSLs. Most importantly, the magnetic response, calculated with error-controlled numerical methods, shows precise signatures of a rank-2 U(1) gauge theory, providing compelling evidence of a fracton QSL. Our results also reveal the distinct fingerprints of gapless gauge excitations, manifested in power-law spin correlations. Remarkably, this constitutes a realization of photons in 2+1 spacetime dimensions, previously thought impossible due to instanton proliferation^46^. As is typical for fracton systems, our model shows strong Hilbert space fragmentation, giving rise to macroscopically many dynamically disconnected subspaces even in the fracton-free sector of the Hilbert space, see also Supplementary Material for details. While such effects are detrimental to fractonic quantum properties in the spin-1/2 version of our model^47^, we find that in the spin-1 case, Hilbert space fragmentation can be helpful in this respect. Particularly, in addition to a fracton QSL in the system’s ground state sector, we identify the same phase in generic excited sectors where its region of stability as a function of model parameters is even increased. The robustness of an emergent rank-2 U(1) gauge theory across different energy scales of our model may be helpful for experimental realizations of our theoretical predictions, e.g., on the basis of synthetic Rydberg atom platforms^48–50^.

## Results

### Model

**Fig. 1:**
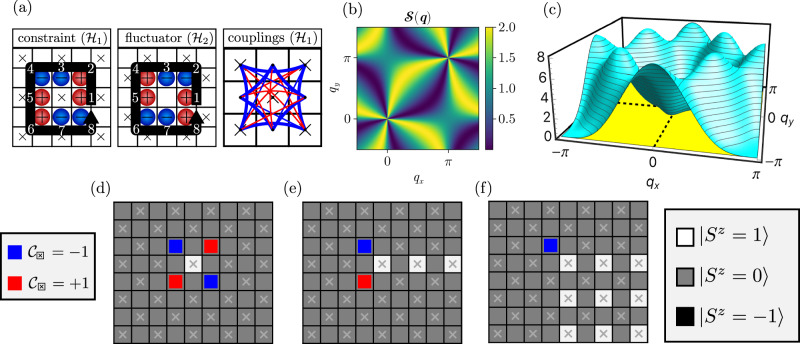
Definition and basic properties of the spiderweb model. **a** Sign structure of the ground state constraints and fluctuators on eight-site clusters around sublattice 1 and sublattice 2 sites, respectively. The right panel shows the couplings that follow from squaring the constraint. Blue (red) couplings correspond to *J*_*ij*_ = −1 (*J*_*ij*_ = 1), and thick lines indicate interactions with ∣*J*_*ij*_∣ = 2. **b** Spin structure factor in Gaussian approximation featuring fourfold pinch points. **c** Band structure of the coupling matrix *J*_*ij*_ of ${{{\mathcal{H}}}}_{1}$ for *J* = 1 with a dispersive upper band, flat lower band and quartic band touching points at ***q*** = (0, 0) and ***q*** = (*π*, *π*). **d** Acting with ${S}_{i}^{+}$ on a defect-free vacuum state $\left\vert 0,\ldots,0\right\rangle$ creates a quadrupole of four fractons with positive (red squares) and negative (blue squares) charges. **e** A *lineon*, a dipole of fractons, which may be moved along the *x*-axis by single spin flips on the ${\hskip-0.1em{\Box}\hskip-0.99em\times}$ sites. **f** Isolated fracton at the corner of the boundary between two distinct domains.

We define our model on the square lattice, where we use the convention that the spins reside at the centers of the squares. For the definition of our model, we distinguish between the two sublattices of the square lattice, one marked with a cross (${\hskip-0.1em{\Box}\hskip-0.99em\times}$, sublattice 1) and the other drawn as empty squares (□, sublattice 2) as shown in Fig. 1. The Hamiltonian consists of three terms ${{\mathcal{H}}}={{{\mathcal{H}}}}_{1}+{{{\mathcal{H}}}}_{2}+{{{\mathcal{H}}}}_{3}$ given by

1 $$\begin{array}{l}{{{\mathcal{H}}}}_{1}={\displaystyle\frac{J}{2}}{\sum }_{\hskip-0.1em{\Box}\hskip-0.7em\times}{{{\mathcal{C}}}}_{\hskip-0.1em{\Box}\hskip-0.7em\times}^{2},\hfill\\ {{{\mathcal{H}}}}_{2}=-{J}^{{\prime} }{\sum }_{\square }\left({{{\mathcal{F}}}}_{\square }+{{{\mathcal{F}}}}_{\square }^{{\dagger} }\right),\hfill\\ {{{\mathcal{H}}}}_{3}=\mu {\sum }_{\square }\left({{{\mathcal{F}}}}_{\square }^{{\dagger} }{{{\mathcal{F}}}}_{\square }+{{{\mathcal{F}}}}_{\square }{{{\mathcal{F}}}}_{\square }^{{\dagger} }\right)\end{array}$$

with

2 $${{{\mathcal{C}}}}_{\hskip-0.1em{\Box}\hskip-0.7em\times} 	={S}_{{\hskip-0.1em{\Box}\hskip-0.7em\times}_{1}}^{z}+{S}_{{\hskip-0.1em{\Box}\hskip-0.7em\times}_{2}}^{z}-{S}_{{\hskip-0.1em{\Box}\hskip-0.7em\times}_{3}}^{z}-{S}_{{\hskip-0.1em{\Box}\hskip-0.7em\times}_{4}}^{z}+{S}_{{\hskip-0.1em{\Box}\hskip-0.7em\times}_{5}}^{z}+{S}_{{\hskip-0.1em{\Box}\hskip-0.7em\times}_{6}}^{z}-{S}_{{\hskip-0.1em{\Box}\hskip-0.7em\times}_{7}}^{z}-{S}_{{\hskip-0.1em{\Box}\hskip-0.7em\times}_{8}}^{z},\\ {{{\mathcal{F}}}}_{\square } 	={S}_{{\square }_{1}}^{+}{S}_{{\square }_{2}}^{-}{S}_{{\square }_{3}}^{-}{S}_{{\square }_{4}}^{+}{S}_{{\square }_{5}}^{+}{S}_{{\square }_{6}}^{-}{S}_{{\square }_{7}}^{-}{S}_{{\square }_{8}}^{+}.$$

In this paper, ${S}_{i}^{z}$, ${S}_{i}^{+}$ and ${S}_{i}^{-}$ are spin-1 operators, and we refer to our companion paper^47^ for an investigation of the spin-1/2 model. Note the site labeling convention where □_*a*_ (${\hskip-0.1em{\Box}\hskip-0.99em\times}$_*a*_) with *a* = 1,…, 8 stands for one of the eight sites adjacent to □ (${\hskip-0.1em{\Box}\hskip-0.99em\times}$), along horizontal, vertical and diagonal directions. Specifically, as illustrated in Fig. 1a, the site □_1_ is located to the right of □ and □_2_, □_3_, … progress counterclockwise around □ (and the same for the sites ${\hskip-0.1em{\Box}\hskip-0.99em\times}$_*a*_). If no index *a* is provided, as e.g. for ${{{\mathcal{C}}}}_{\hskip-0.1em{\Box}\hskip-0.7em\times}$, the quantity is located directly at site ${\hskip-0.1em{\Box}\hskip-0.99em\times}$. Furthermore, *i* denotes a general site index not specifying the sublattice.

The sign structure + + − − + + − − of the spin sums in ${{{\mathcal{C}}}}_{\hskip-0.1em{\Box}\hskip-0.7em\times}$ and of the raising/lowering operators in ${{{\mathcal{F}}}}_{\square }$ are depicted in Fig. 1a and have close similarities. We call our model the *spiderweb model* since factoring out the squares in ${{{\mathcal{C}}}}_{\hskip-0.1em{\Box}\hskip-0.7em\times}^{2}$ gives rise to a network of spin interactions resembling a spiderweb, see Fig. 1a.

The general structure of ${{\mathcal{H}}}$ consisting of three terms is common to many spin liquid or quantum dimer models^6,8,51–53^. The first term ${{{\mathcal{H}}}}_{1}$ defines a low-energy subspace spanned by states that fulfill the constraints ${{{\mathcal{C}}}}_{\hskip-0.1em{\Box}\hskip-0.7em\times}=0$ for the eight-site clusters around all ${\hskip-0.1em{\Box}\hskip-0.99em\times}$, the so-called *constrained subspace*. The number of these states scales exponentially with the total number of sites (which also applies to the corresponding spin-1/2 model^47^), see Supplementary Material for details. The second term ${{{\mathcal{H}}}}_{2}$ induces tunneling between states in the constrained subspace. Importantly, the so-called *fluctuators* ${{{\mathcal{F}}}}_{\square }$ commute with each of the constraint operators ${{{\mathcal{C}}}}_{\hskip-0.1em{\Box}\hskip-0.7em\times}$, such that the quantum dynamics generated by ${{{\mathcal{H}}}}_{2}$ does not lead the system out of the constrained subspace.

While ${{{\mathcal{H}}}}_{1}$ consists of usual two-body spin interactions, the eight-site ring-exchange term ${{{\mathcal{F}}}}_{\square }$ might first seem complicated and artificial. However, this term represents the shortest product of spin flip operators that generates tunneling between states within the constrained subspace. This implies that *any* small perturbation of ${{{\mathcal{H}}}}_{1}$ that is off-diagonal in the Ising ${S}_{i}^{z}$ basis will inevitably generate ${{{\mathcal{H}}}}_{2}$ when projected onto the constrained subspace. For example, ${{{\mathcal{H}}}}_{2}$ is generated in fourth order perturbation theory for small transverse nearest neighbor couplings ${S}_{i}^{x}{S}_{j}^{x}+{S}_{i}^{y}{S}_{j}^{y}$ or in eighth order perturbation theory in small transverse magnetic fields $\sim {S}_{i}^{x}$. These properties are similar to quantum spin ice, where minimal hexagon loop moves are generated in third-order perturbation theory in transverse nearest neighbor couplings^6,8^.

The third term ${{{\mathcal{H}}}}_{3}$ counts the number of eight-site clusters that are not annihilated by ${{{\mathcal{F}}}}_{\square }$ or ${{{\mathcal{F}}}}_{\square }^{{\dagger} }$. It can be understood as a chemical potential for flippable clusters and has been introduced in many other spin liquid models before, such as short-range ${{\mathbb{Z}}}_{2}$^51,53,54^ or algebraic pyrochlore U(1) spin liquids^6,8,51,52^. By changing *μ* the system can be tuned through different phases. Specifically, the limits *μ* → *∞* (*μ* → −*∞*) where the numbers of flippable clusters are minimized (maximized) typically give rise to simple ordered states. On the other hand, at $\mu \in (0,{J}^{{\prime} }]$ the competition between kinetic (${{{\mathcal{H}}}}_{2}$) and potential (${{{\mathcal{H}}}}_{3}$) energy may create non-trivial strongly fluctuating quantum phases. The system with $\mu={J}^{{\prime} } > 0$, known as the Rokhsar-Kivelson point^55^, is exactly solvable, where the ground state is an equal weight superposition of all states in the constrained subspace. More precisely, if the constrained subspace is again divided into dynamically disconnected sectors by the action of ${{{\mathcal{F}}}}_{\square }$ and ${{{\mathcal{F}}}}_{\square }^{{\dagger} }$ (as is the case in our system), a ground state can be constructed from the equal weight superposition of states in *each* of these dynamically disconnected sectors.

For large enough sectors, such a massive superposition can be associated with a quantum spin liquid. However, whether this spin liquid also exists as an extended phase for $\mu < {J}^{{\prime} }$ is highly non-trivial and model dependent.

### General fractonic properties

Our comprehensive numerical investigations presented below show that the spin-1 spiderweb model exhibits a fracton QSL in a finite region in *μ*. These studies can be understood as a continuation of our investigations of the corresponding spin-1/2 model in our companion paper^47^. However, that work revealed that the quantum dynamics induced by ${{{\mathcal{H}}}}_{2}$ are too weak to generate a QSL. The reason for the suppressed quantum dynamics in the spin-1/2 case is a severe Hilbert space fragmentation as is typical for fracton models^56–58^. Specifically, ${{{\mathcal{H}}}}_{2}$ splits up the constrained subspace into an even finer structure of many dynamically disconnected subspaces, each with only trivial quantum dynamics. This results in either long-range ordered phases or *classical fracton spin liquids*, i.e., ground states characterized by a classical rank-2 gauge theory without coherent quantum dynamics. As we will see below, the availability of more spin states ${S}_{i}^{z}\in \{-1,0,1\}$ for spin-1 significantly enhances the quantum dynamics and promotes fractonic properties at a quantum level. Note, however, that Hilbert space fragmentation is still present in the spin-1 model and again gives rise to a vast number of dynamically isolated subspaces scaling exponentially in the number of sites, as discussed in the Supplementary Material.

While the identification of a fracton QSL requires advanced numerical approaches, some general fractonic properties of the spiderweb model are already evident from its structure and are independent of the spin magnitude. Here, we explain the key properties and refer to our companion paper^47^ for a more in-depth discussion.

#### Gaussian approximation

The Gaussian approach^40,42,43^ consists of treating the spins as unconstrained variables (no local normalization or quantization imposed) and in Fourier space ${S}_{m}^{z}({{\boldsymbol{q}}})={\sum}_{i\in m}{e}^{\imath {{\boldsymbol{q}}}\cdot {{{\boldsymbol{r}}}}_{i}}{S}_{i}^{z}$ where *m* = 1, 2 denotes the two sublattices. Expanded in lowest non-vanishing order (quadratic order) around ***q*** = 0 the classical constraints ${{{\mathcal{C}}}}_{\hskip-0.1em{\Box}\hskip-0.7em\times}=0$ then take the form

3 $$({q}_{x}^{2}-{q}_{y}^{2}){S}_{2}^{z}({{\boldsymbol{q}}})+4{q}_{x}{q}_{y}{S}_{1}^{z}({{\boldsymbol{q}}})=0\ .$$

Arranging the Fourier-components in a symmetric and trace-free matrix $\underline{S}({{\boldsymbol{q}}})$ defined as

4 $$\underline{S}({{\boldsymbol{q}}})=\left(\begin{array}{ll}{S}_{2}^{z}({{\boldsymbol{q}}})&2{S}_{1}^{z}({{\boldsymbol{q}}})\\ 2{S}_{1}^{z}({{\boldsymbol{q}}})&-{S}_{2}^{z}({{\boldsymbol{q}}})\end{array}\right)$$

the constraint can be written compactly as ${q}_{\mu }{q}_{\nu }{\underline{S}}^{\mu \nu }({{\boldsymbol{q}}})=0$ which is exactly the momentum space version of the generalized charge-free Gauss’ law of a trace-free rank-2 U(1) electrostatic theory, ∂_*μ*_∂_*ν*_*E*^*μν*^ = 0, with a fictitious matrix-valued ‘electric’ field *E*^*μν*^^10,11,13,15,59^ (see also the Supplementary Material for details on the derivation of the Gauss law). Violations of the constraint ∂_*μ*_∂_*ν*_*E*^*μν*^ = *ρ* ≠ 0 take the role of charges, which in this higher-rank case are called (scalar type-I) fractons with no mobility in real space. We note that an identical rank-2 Gauss’ law also follows from an expansion of the constraint around ***q*** = (*π*, *π*), indicating that long-wavelength fluctuations around ferromagnetic and antiferromagnetic states are both of fractonic nature. As explained in ref. ^47^, the emergent rank-2 Gauss’ laws are a direct consequence of the special sign structure of the spin sum in ${{{\mathcal{C}}}}_{\hskip-0.1em{\Box}\hskip-0.7em\times}$ corresponding to discretized second derivatives.

The rank-2 Gauss’ law manifests in singular points in the spin structure factor

5 $${{\mathcal{S}}}({{\boldsymbol{q}}})=\frac{1}{{N}_{{{\rm{sites}}}}}{\sum}_{mn}\langle {S}_{m}^{z}(-{{\boldsymbol{q}}}){S}_{n}^{z}({{\boldsymbol{q}}})\rangle$$

at *T* = 0 known as fourfold pinch points^59^. Within the present Gaussian formulation these features are obtained by carrying out 〈 ⋯ 〉 as a projection onto the Fourier-modes that fulfill Eq. (3). The result shown in Fig. 1b features clear fourfold pinch points in the extended Brillouin zone *q*_*x*_, *q*_*y*_ ∈ [0, 2*π*) at ***q*** = (0, 0) and ***q*** = (*π*, *π*) which are just the points where an expansion of the constraint has the form of a rank-2 Gauss’ law.

An alternative description of the same properties is obtained by writing ${{{\mathcal{H}}}}_{1}$ as a 2 × 2 matrix in the Fourier-components ${S}_{1}^{z}({{\boldsymbol{q}}})$ and ${S}_{2}^{z}({{\boldsymbol{q}}})$. As shown in Fig. 1c, the diagonalization of this matrix results in a flat bottom band (describing the constrained subspace) which is connected to an upper dispersive band at two band touching points, which occur at the exact momenta of the pinch points. The fourfold nature of pinch points implies that the dispersive band is *quartic* in ***q*** in the vicinity of the band touching points.

#### Classical fracton configurations from ${{{\mathcal{H}}}}_{1}$

A particularly intuitive access to the fractonic properties of the spiderweb model is obtained by examining classical fracton configurations in real space, as shown in Fig. 1d–f. In Fig. 1d, a single ${S}_{i}^{z}=1$ site on sublattice 1 in a homogeneous ${S}_{i}^{z}=0$ background (which is one possible ground state of ${{{\mathcal{H}}}}_{1}$) corresponds to four violated constraints ${{{\mathcal{C}}}}_{\hskip-0.1em{\Box}\hskip-0.7em\times}\ne 0$ (fractons) forming a quadrupole. This implies that a single spin flip fractionalizes into four fractons, directly demonstrating that fractionalization – a hallmark of a QSL – is already intrinsic to ${{{\mathcal{H}}}}_{1}$. These four violated constraints constitute the smallest group of fractons that can move freely as a whole by lowering the changed spin back to zero and raising a neighboring one. On the other hand, a semi-infinite string of ${S}_{i}^{z}=1$ sublattice 1 sites [Fig. 1e] gives rise to a dipole of fractons (so-called lineons^60^) which can move perpendicular but not parallel to its dipole moment. Finally, a corner in the domain wall of Fig. 1f corresponds to an isolated fracton that is completely immobile, unless infinitely many spins are flipped (moving the lower right domain as a whole) or additional fractons are created. This immobility is a direct consequence of the dipole conservation, which is again a result of the emergent rank-2 Gauss’ law^13^.

#### Conserved magnetizations

**Fig. 2:**
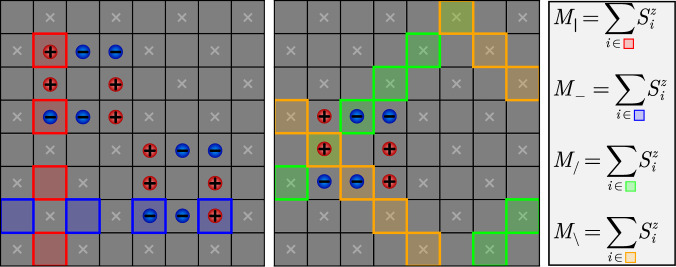
String-like magnetizations *M*_*∣*_, *M*_*-*_, *M*_*\*_, *M*_*/*_ which are conserved under the application of the fluctuator ${{{\mathcal{F}}}}_{\square }$. Colored squares correspond to the sites that are summed over. Also shown is the action of ${{{\mathcal{F}}}}_{\square }$ at exemplary locations which always cause canceling contributions to any of the operators *M*_***∣***_, *M*_***-***_, *M*_***\***_, and *M*_***/***_.

Typical fracton properties are also reflected in the constants of motion of our spiderweb model. From the definition of ${{{\mathcal{F}}}}_{\square }$ in Eq. (2), also shown in Fig. 1a, it can be seen that the total magnetization ${M}_{m}^{z}={\sum}_{i\in m}{S}_{i}^{z}$ in each sublattice *m* = 1, 2 and the (spin) dipole moment $\sim {\sum}_{i}{{{\boldsymbol{r}}}}_{i}{S}_{i}^{z}$ are conserved. In addition to these global conserved magnetizations, the system also has subdimensional constants of motion, as they are characteristic of type-I fracton models. In our case, they correspond to spin sums *M*_***\***_, *M*_***/***_(*M*_***∣***_, *M*_***-***_) on diagonal (straight) lines containing only sites of sublattice 1 (sublattice 2), see Fig. 2. These subdimensional conserved magnetizations can be viewed in analogy to the subsystem symmetries known from gapped fracton models^18,61–63^.

### Green Function Monte Carlo results and gauge theory

#### Ground state properties

We shall now discuss the ground state quantum properties of Eq. (1), in the limit ${J}^{{\prime} }\ll J$, where we need to only consider fracton-free sectors for which ${{{\mathcal{H}}}}_{1}=0$. For ${J}^{{\prime} } > 0$, our model is free of the sign problem, and thus observables may be obtained numerically within statistical error bars via quantum Monte Carlo (QMC). As we are interested in ground state properties, we choose the so-called Green function Monte Carlo (GFMC) method, which allows sampling of ground state observables within a given ergodicity sector of ${{\mathcal{H}}}$^64,65^. Details regarding this method are summarized in Section IV A.

**Fig. 3:**
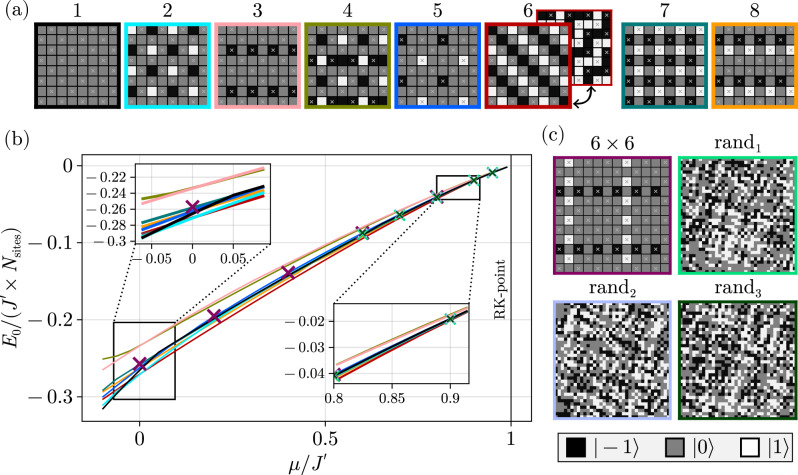
Low-energy sectors of the spin-1 spiderweb model. **a** Maximally flippable configurations defining the eight most energetically favorable sectors with a 4 × 4 unit cell, so-called parent states. **b** Energy of each sector shown in (**a**) (solid lines) and (**c**) (markers) as a function of *μ*. **c** Other configurations that define individual sectors, such as a configuration with a 6 × 6 unit cell, as well as randomized configurations. For the configurations in (**a**), system sizes of *L* = 20 are used, while for (**c**), the system sizes are *L* = 24 for the 6 × 6 sector and *L* = 36 for the random sectors.

To identify the sector containing the ground state out of an exponentially large number of sectors, we first generate *all* periodic fracton-free spin configurations (in *S*^*z*^ basis) with a 4 × 4 unit cell. Our numerical simulations are then performed on larger *L* × *L* systems (with *L* up to 36), where the starting configurations correspond to periodic tilings of the *L* × *L* system with these 4 × 4 unit cells. Determining the ground states in the sectors connected to each of these 4 × 4 ‘parent’ states, we systematically scan large numbers of subspaces. Specifically, discarding configurations with no flippable clusters and eliminating redundant configurations using point-group or time reversal symmetries, we find 1104 periodic 4 × 4 configurations. Under the application of ${{{\mathcal{H}}}}_{2}$ these configurations give rise to 28 fully inequivalent, disconnected Hilbert space sectors, which can be distinguished via their energetic properties and their maximally flippable configuration, which maximizes ${\sum}_{\square }{{{\mathcal{F}}}}_{\square }^{{\dagger} }{{{\mathcal{F}}}}_{\square }+{{{\mathcal{F}}}}_{\square }{{{\mathcal{F}}}}_{\square }^{{\dagger} }$. These configurations are depicted for the eight lowest energetic sectors in Fig. 3a. Clearly, this approach does not capture all Hilbert space sectors. For example, it is in principle possible that the ground state lies in another sector connected to periodic 6 × 6 tilings (however, an exhaustive enumeration of such tilings proved to be numerically infeasible due to the large number of solutions). In that case, however, already simulations of sectors from periodic 4 × 4 states are expected to show traces of 6-site periodic correlations such as Bragg peaks in the spin structure factor at momenta ***q*** = 2*π*(1/6, 1/6). This particularly applies to the regime $\mu \ll {J}^{{\prime} }$ where systems tend to establish long-range order built from periodic tilings. The absence of such Bragg peaks in our results for 4 × 4 parent states indicates that our approach includes enough sectors to identify the overall ground state.

In Fig. 3b, we show the energy of the eight energetically lowest-lying sectors as a function of *μ*, where Fig. 3a illustrates the corresponding eight 4 × 4 periodic parent states. Figure 3b also displays the energies of other sectors shown in Fig. 3c, such as one with a larger 6 × 6 unit cell, as well as randomly sampled fracton-free configurations without any periodic parent states.

The most interesting regime is at $0 < \mu \le {J}^{{\prime} }$ where the competition between kinetic and potential energy increases quantum fluctuations. Here, we find that the system’s ground state lies in the sector of the diagonal stripe state [shown as sector number 6 (foreground) in Fig. 3a]. Interestingly, this sector also contains the state shown in the background of sector number 6 in Fig. 3a, which has a staircase-pattern built from local spin states ${S}_{i}^{z}=\pm 1$. This configuration is an analogue of the staircase state discussed in ref. ^47^ in the context of the spin-1/2 spiderweb model, where the ground state consists of fluctuations around a staircase configuration with ${S}_{i}^{z}=\pm 1/2$.

Surprisingly, the sector of the homogeneous ${S}_{i}^{z}=0$ state [number 1 in Fig. 3a] is an excited sector across the entire regime $0\le \mu < {J}^{{\prime} }$ even though it allows for substantial quantum fluctuations: This state has the unique property that all clusters □ are flippable by ${{{\mathcal{F}}}}_{\square }$ and by ${{{\mathcal{F}}}}_{\square }^{{\dagger} }$ and thus exhibits the absolute maximum number of flippable clusters among all spin-1 Ising configurations. With this property, it is clear that this sector must become the ground state sector for sufficiently small *μ*. Our results in Fig. 3b confirm this to be the case for *μ* ≲ 0. With decreasing *μ*, the negative potential term suppresses fluctuations into local ${S}_{i}^{z}=\pm 1$ configurations and in the limit *μ* → −*∞* the ground state continuously transforms into the exact homogeneous ${S}_{i}^{z}=0$ state. Thus, even though no symmetry breaking occurs in this regime and no long-range order is observed, the system is a trivial paramagnet that is continuously connected to a product state (${S}_{i}^{z}=0$ on every site).

In the other limit $\mu > {J}^{{\prime} }$, ${{\mathcal{H}}}$ is positive semi-definite. There, the exact ground state is given by trivial sectors containing only a single configuration with no flippable clusters, which is annihilated by ${{\mathcal{H}}}$. While there is still a substantial number of these sectors, it is clear that no quantum dynamics can occur in the ground state for $\mu > {J}^{{\prime} }$ and the system behaves classically.

Having identified the ground state sector at $0\le \mu < {J}^{{\prime} }$, it remains to be determined whether the long-range correlations in its diagonal stripe parent state can be wiped out by quantum fluctuations giving rise to a spin liquid. Even at the RK point $\mu={J}^{{\prime} }$ where the exact wave function is an equal weight superposition of all states in that sector, it is not a priori clear whether a QSL forms. Indeed, for spin *S* = 1/2, it was found that the large degree of Hilbert space fragmentation competes with quantum dynamics and leads to strongly classical behavior with negligible impact of quantum fluctuations even at the RK-point^47^. Besides this, spin liquids in two-dimensional quantum dimer models are often found to have a vanishing stability regime, such that the RK point behaves as a quantum critical point between two ordered phases, for example, on bipartite lattices^55,66–68^.

**Fig. 4:**
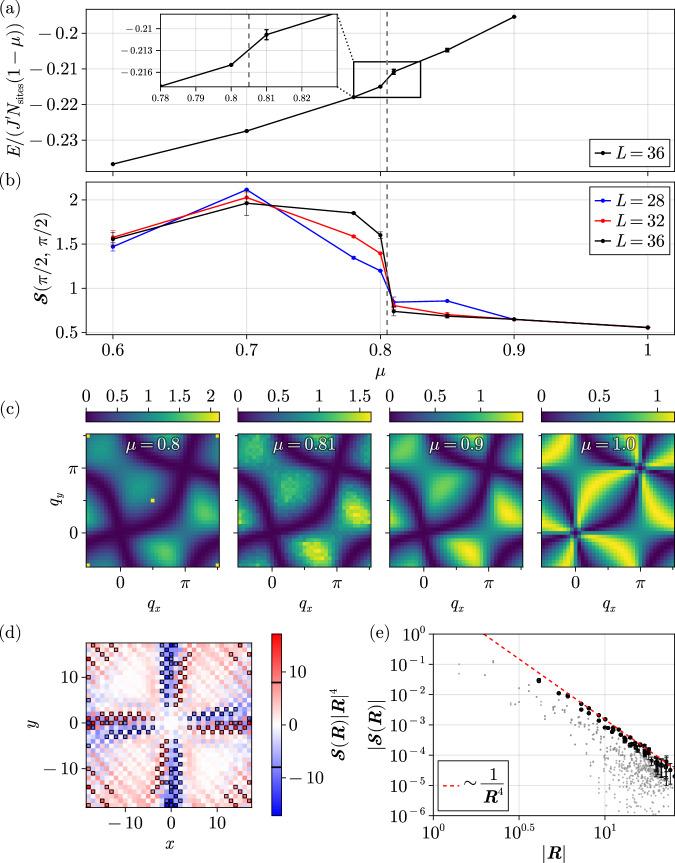
Ground state properties of the spin-1 spiderweb model. **a** Energy per site in the diagonal stripe sector scaled by ${({J}^{{\prime} }-\mu )}^{-1}$. The dashed line indicates the location of the energy’s discontinuity. **b** Ground state spin structure factor [Eq. (5)] in the diagonal stripe sector from GFMC at ***q*** = (*π*/2, *π*/2) as a function of *μ* for different system sizes *L*. **c** Momentum resolved ground state spin structure factor in the diagonal stripe sector from GFMC for different *μ* and *L* = 36. **d** Direct space spin correlations ${{\mathcal{S}}}({{\boldsymbol{R}}})$ for $\mu=0.9{J}^{{\prime} }$, scaled by ∣***R***∣^4^, with dominant correlations highlighted in black. **e** Decay of spin correlations as a function of distance ∣***R***∣ compared to a power-law  ~ ∣***R***∣^−4^ (red dashed line) for $\mu=0.9{J}^{{\prime} }$. Gray (black) markers indicate generic (dominant) correlations.

In investigation of this, Fig. 4a shows an enlarged view of the energy per site in the diagonal stripe sector as a function of *μ*, rescaled by a factor of ${({J}^{{\prime} }-\mu )}^{-1}$ for better visibility of features beside the trivial energetic scaling $\sim -({J}^{{\prime} }-\mu )$. A kink in the energy around $\mu=0.8{J}^{{\prime} }$ indicates a first-order phase transition. To distinguish these phases, Fig. 4b shows the value of the spin structure factor at ***q*** = (*π*/2, *π*/2), the momentum of the diagonal stripe order as a function of *μ*. Here, a much stronger discontinuity can be seen between $\mu=0.8{J}^{{\prime} }$ and $\mu=0.81{J}^{{\prime} }$ which becomes increasingly pronounced with the system size. Inspecting the full momentum dependence of ${{\mathcal{S}}}({{\boldsymbol{q}}})$ in Fig. 4c gives further insight into the nature of the two phases. For $\mu \le 0.8{J}^{{\prime} }$, the spin structure factor shows a clear peak at ***q*** = (*π*/2, *π*/2), signaling conventional magnetic long-range order. For $\mu > 0.8{J}^{{\prime} }$, this peak disappears abruptly, revealing fourfold pinch points that are suppressed around their respective centers located at ***q*** = (0, 0) and ***q*** = (*π*, *π*). Indeed, this pinch point suppression is a well-known signature of emergent photons in conventional U(1) gauge theories and has been numerically confirmed for quantum spin ice^8^. As *μ* increases, the intensity around the pinch point is continuously restored, and, finally, at the RK point precisely corresponds to the Gaussian approximation from Fig. 1b (Note that the  + -shaped minima at the pinch point origin are finite-size effects which always correspond to the four momenta closest to the pinch point at any system size *L* with their volume shrinking to zero for *L* → *∞*). We emphasize that the appearance of a fourfold pinch point within a single sector of the Hilbert space is in contrast to spin-1/2, where this feature only appears after summing classical contributions from many sectors^47^. Figure 4d displays the directional dependence of the real space correlation function ${{\mathcal{S}}}({{\boldsymbol{R}}})=\frac{1}{N}{\sum}_{i}\langle {S}^{z}({{{\boldsymbol{r}}}}_{i}){S}^{z}({{\boldsymbol{R}}}-{{{\boldsymbol{r}}}}_{i})\rangle$ at $\mu=0.9{J}^{{\prime} }$. The rescaling by ∣***R***∣^4^ highlights a checkerboard pattern of weak and strong correlations with dominant directions near the Cartesian axes. Figure 4e shows the distance dependence of the correlation function, indicating a power-law ~∣***R***∣^−4^ behavior consistent with a gapless state^69^ (see Supplementary Material for an explanation of the ∣***R***∣^−4^ scaling).

These findings provide strong evidence of a gapless QSL at the RK point, which remains stable in a finite region $0.81{J}^{{\prime} }\le \mu \le {J}^{{\prime} }$. Later, this result will be further substantiated using more rigorous field theory arguments.

A peculiar feature of Fig. 4c is the rotational asymmetry around the pinch points. Specifically, at $\mu < {J}^{{\prime} }$, the 90^∘^ rotation symmetry of a perfect fourfold pinch point is lowered to a two-fold symmetry, giving rise to two of four lobes with relatively higher intensity. Towards the RK point $\mu={J}^{{\prime} }$, this fourfold rotational symmetry is continuously restored. The rotational asymmetry at $\mu < {J}^{{\prime} }$ is a consequence of the fact that the diagonal stripe configuration itself breaks 90^∘^ rotation symmetry, which is not restored by quantum fluctuations. In fact, it can be checked that a simulation starting from a 90° rotated diagonal stripe state yields a 90° rotated spin structure factor. This implies that the symmetry breaking is *not spontaneous* but rather imposed by the considered parent state. This latter property is further substantiated in the Supplementary Material where it is shown that 90° rotated versions of the diagonal stripe configuration lie in independent, dynamically disconnected sectors. Therefore, the broken rotational symmetry is not contradictory to a QSL. In fact, in the next section, we will show that it is fully compatible with a spin liquid obeying an emergent rank-2 U(1) gauge structure.

#### Emergent rank-2 U(1) quantum electrodynamics

The results above indicate that the spin-1 spiderweb model realizes a stable QSL phase for $0.8{J}^{{\prime} } < \mu \le {J}^{{\prime} }$. Characterized by quantum fluctuations within a manifold of configurations satisfying a rank-2 Gauss’ law, this phase is thus expected to be a higher-rank U(1) QSL. To further verify this expectation, we turn to an effective field theoretical description of Eq. (1). Indeed, the well-known mapping of quantum spin ice to a U(1) gauge theory^6–8^ can be transferred to our higher-rank system. Following a similar strategy, we express the spin operators ***S***_*i*_ in terms of conjugate rotor variables ${A}_{i}^{xy/xx}$ and ${E}_{i}^{xy/xx}$ via

6 $${S}_{i}^{\pm }=\left\{\begin{array}{ll}\sqrt{2}{e}^{\pm {\imath} {A}_{i}^{xy}}\quad &{{i}}=\,{{\mbox{center}}} \, {{\rm{of}}}\,{\hskip-0.1em{\Box}\hskip-0.99em\times}\, ({{\mbox{sublattice 1}}})\\ \sqrt{2}{e}^{\pm {\imath} {A}_{i}^{xx}}\quad &{{i}}=\,{{\mbox{center}}} \, {{\rm{of}}}\,\square \,({{\mbox{sublattice 2}}})\end{array}\right.,$$

7 $${S}_{i}^{z}=\left\{\begin{array}{ll}{E}_{i}^{xy}\quad & {{i}}={{\mbox{center}}} \, {{\rm{of}}}\,{\hskip-0.1em{\Box}\hskip-0.99em\times}\, ({{\mbox{sublattice 1}}})\\ {E}_{i}^{xx}\quad & {{i}}={{\mbox{center}}} \, {{\rm{of}}}\,\square \,({{\mbox{sublattice 2}}})\,\end{array}\right.,$$

with the commutation relations $[{A}_{i}^{xy},{E}_{j}^{xy}]=[{A}_{i}^{xx},{E}_{j}^{xx}]=\imath {\delta }_{ij}$ and $[{A}_{i}^{xy},{E}_{j}^{xx}]=[{A}_{i}^{xx},{E}_{j}^{xy}]=0$. Note that the factor $\sqrt{2}$ in Eq. (6) accounts for the spin-1 quantum number. As conjugate rotor variables, the fields ${A}_{i}^{xy/xx}\in [0,2\pi ]$ are compact while the ‘number operators’ ${E}_{i}^{xy/xx}\in {\mathbb{Z}}$ are integer, and can thus be different from −1, 0, 1 in principle. Furthermore, ${A}_{i}^{xy/xx}$ correspond to the components of a 2 × 2 trace-free (${A}_{i}^{xx}=-{A}_{i}^{yy}$) and symmetric (${A}_{i}^{xy}={A}_{i}^{yx}$) matrix-valued field that takes the role of a rank-2 generalization of the vector potential in conventional electrodynamics. Similarly, ${E}_{i}^{xy/xx}$ are the components of a matrix-valued electric field with the analogous properties ${E}_{i}^{xx}=-{E}_{i}^{yy}$ and ${E}_{i}^{xy}={E}_{i}^{yx}$. We may now define a gauge invariant emergent magnetic field *B*_□_ (see Supplementary Material for details) for each center of a □ cluster via

8 $${B}_{\square }={A}_{{\square }_{1}}^{xy}-{A}_{{\square }_{2}}^{xx}-{A}_{{\square }_{3}}^{xy}+{A}_{{\square }_{4}}^{xx}+{A}_{{\square }_{5}}^{xy}-{A}_{{\square }_{6}}^{xx}-{A}_{{\square }_{7}}^{xy}+{A}_{{\square }_{8}}^{xx},$$

where the sign pattern follows that of the fluctuator [Fig. 1a] and the notation for sites *i* = □_*a*_ is the same as in Eq. (1).

With these ingredients we can now formulate an effective field theory for ${{\mathcal{H}}}$ in the constrained subspace,

9 $${{{\mathcal{H}}}}_{{{\rm{eff}}}}=\frac{U}{2}\left[{\sum}_{\hskip-0.1em{\Box}\hskip-0.7em\times}{({E}_{\hskip-0.1em{\Box}\hskip-0.7em\times}^{xy})}^{2}+{\sum}_{\square }{({E}_{\square }^{xx})}^{2}\right]\\+\frac{K}{2}{\sum}_{\square }{B}_{\square }^{2}+\frac{W}{2}{\sum}_{\square }{{{\mathcal{N}}}}_{\square }^{2},$$

with

10 $${{{\mathcal{N}}}}_{\square }={E}_{{\square }_{1}}^{xy}-{E}_{{\square }_{2}}^{xx}-{E}_{{\square }_{3}}^{xy}+{E}_{{\square }_{4}}^{xx}+{E}_{{\square }_{5}}^{xy}-{E}_{{\square }_{6}}^{xx}-{E}_{{\square }_{7}}^{xy}+{E}_{{\square }_{8}}^{xx}.$$

The first term ~*U* suppresses high values of $\left| {E}^{xy/xx}_{i}\right|$ to (approximately) enforce the constraint ${S}_{i}^{z}=-1,0,1$. This term describes the energy density of the electric field and has the same form as the corresponding term in a usual Maxwellian field theory.

The second term  ~ *K* comes from inserting Eq. (6) into ${{{\mathcal{H}}}}_{2}$ and using Eq. (8). This gives ${{{\mathcal{H}}}}_{2} \sim -\cos {B}_{\square }$ and, when expanded to quadratic order, yields $\sim {B}_{\square }^{2}$, corresponding to the energy density of the magnetic field, as in a Maxwellian field theory. The assumption behind the expansion that *B*_□_ fluctuates only mildly around *B*_□_ = 0, however, is not necessarily fulfilled. In fact, phase slip events between two minima of the cosine *B*_□_ → *B*_□_ + 2*π* that cannot be captured within a finite-order expansion are a known phenomenon of U(1) field theories in 2 + 1 dimensional spacetime^46^. If these so-called *instanton* events proliferate, they may drive the system into an ordered and confined phase. By comparing our numerical results for the spin-1 spiderweb model with the predictions of the effective field theory, we will confirm that the assumptions and approximations behind Eq. (9) are justified.

The last term ~*W* in Eq. (9) mimics the potential term ${{{\mathcal{H}}}}_{3}$, where the exact RK point is realized in the limit *U*/*K* → 0 ^8^. Following the construction of refs. ^6,8^, ${{{\mathcal{N}}}}_{\square }$ is obtained from *B*_□_ by replacing *A*^*xy*^ → *E*^*xy*^ and *A*^*xx*^ → *E*^*xx*^ in Eq. (8).

The model in Eq. (9) is a free bosonic theory describing a rank-2 U(1) QSL. Importantly, it can be solved exactly, yielding a single photon mode *ω*(***q***) with gapless nodal points at the pinch point locations. For *U* > 0, away from the RK point, an expansion of *ω*(***q***) in *q* around the pinch points yields in lowest orders

11 $$\omega ({{\boldsymbol{q}}})\approx \sqrt{\frac{KU}{4}}\sqrt{{q}_{x}^{4}+14{q}_{x}^{2}{q}_{y}^{2}+{q}_{y}^{4}}\,$$

revealing a quadratic photon dispersion *ω*(***q***) ~ *q*^2^ for small *q* along any radial direction away from ***q*** = 0. Exactly at the RK point *U* = 0, the photon dispersion becomes even quartic, *ω*(***q***) ~ *q*^4^. The field theory also predicts the spin structure factor ${{\mathcal{S}}}({{\boldsymbol{q}}})$, which we can compare with the numerical results from GFMC by taking the parameters *U*, *K* and *W* as fit parameters [see Supplementary Material for an explicit analytical expression].

In principle, the field theory in Eq. (9) is applicable to any fracton-free Hilbert space sector of the spiderweb model. However, as described in Sec. II C in the context of the diagonal stripe state, specific sectors may imprint a rotational asymmetry. To account for such effects, we add an additional phenomenological term to the Hamiltonian:

12 $${{{\mathcal{H}}}}_{{{{\mbox{eff}}}}} \rightarrow {{{\mathcal{H}}}}_{{{{\mbox{eff}}}}}+\frac{U'}{2}\left[ \sum\limits_{1{{\hskip-0.1em{\Box}\hskip-0.5em\times}}\atop{\,\,\,\,2{{\hskip-0.1em{\Box}\hskip-0.5em\times}}}}(E^{xy}_{1}+E^{xy}_{2})^2+\sum\limits_{1{\Box}\atop{\,\,\,\,2{\Box}}} (E^{xx}_{1}+E^{xx}_{2})^2\right].$$

It corresponds to a modification of the first term in Eq. (9) where the local ${E}_{i}^{2}$-terms are made non-local by coupling electric fields *E*_1_, *E*_2_ on pairs of sites separated along one diagonal direction, and, therefore, breaking 90^∘^ rotation symmetry. Importantly, this gauge-invariant term only affects the directional shape of the emergent photon dispersion and spin structure factor at *small* wavelengths. The long wavelength limit remains unaffected, retaining the exact gapless and 90^∘^ rotation-symmetric photon dispersion of Eq. (11).

To obtain the best fit to these field theory parameters, we define three independent fitting parameters (*A*, *r*, *p*) through $(K,W,U,U^{\prime} )=(4{A}^{2},1,r,pr)$. In the spin structure factor, *A* provides a trivial uniform scaling, while *r* interpolates between the RK point at *r* = 0 and the RK-free limit *r* = *∞*, and $p=U^{\prime} /U$ is the strength of the rotational asymmetry.

**Fig. 5:**
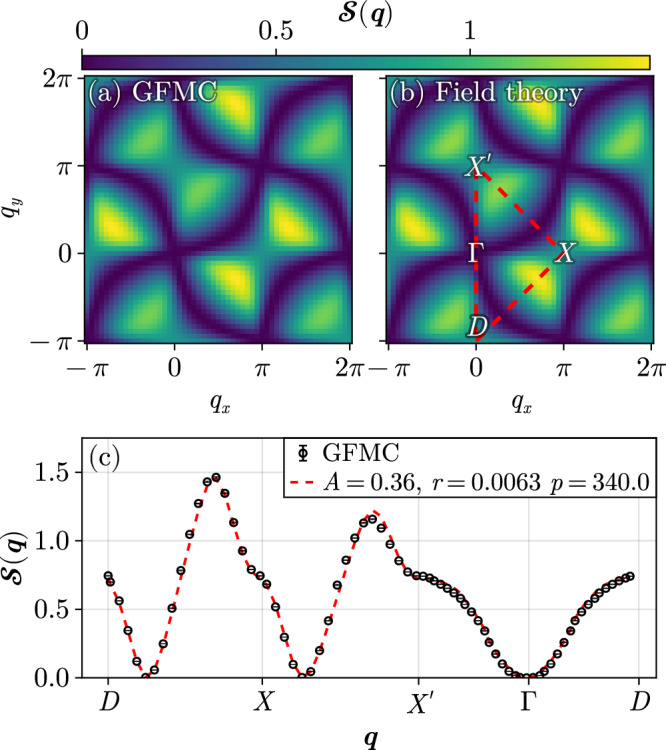
Comparison between ground state spin structure factor and effective field theory. **a** Ground state spin structure factor in the diagonal stripe sector obtained from GFMC at $\mu=0.9{J}^{{\prime} }$ for *L* = 36. **b** Spin structure factor for the best fit to (**a**) using the asymmetric rank-2 U(1) field theory of Eqs. (9, 12). **c** Spin structure factor along the path indicated in (**b**) for the spin model (black empty circles) from (**a**) compared to the field theory fit (red dashed line) from (**b**). Statistical errors are estimated from the standard deviation over 14 independent simulations.

**Fig. 6:**
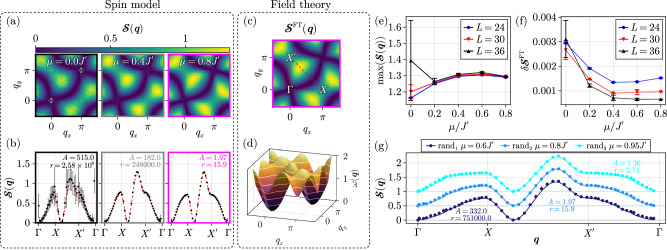
Spin structure factor of excited sectors. **a** Spin structure factor in the 6 × 6 sector [see Fig. 3c] for three different values of *μ* for *L* = 36. **b** Data of (**a**) along line cuts connecting high-symmetry points in momentum space [illustrated in (**c**)]. The red dashed line indicates the best fit to the field theory. **c** Best fit to the field theory for the spin structure factor at $\mu=0.8{J}^{{\prime} }$ shown in (**a**). **d** Photon dispersion as predicted by the field theory for *U* = *K* = 1 and *W* = 0. **e** Maximum of the spin structure factor as a function of *μ* for the 6 × 6 sector and **f** relative deviation $\delta {{{\mathcal{S}}}}^{{{\rm{FT}}}}({{\boldsymbol{q}}})$ to the best fit to the field theory. **g** Line cuts of the spin structure factor in the randomized sectors shown in Fig. 3c for different values of *μ*. Solid lines indicate the best fit to the field theory. For visual clarity, the curve for the rand_2_ (rand_3_) sector is vertically shifted by 0.5 (by 1). In all panels, error bars are estimated by the standard deviation over 14 independent runs.

Figure 5a, b shows the comparison of the spin structure factors from GFMC and from the best fit to the asymmetric field theory, demonstrating nearly perfect agreement. To highlight the excellent quantitative nature of this agreement, in Fig. 5c, we show the spin structure factor for a path along high-symmetry points in reciprocal space, as indicated in Fig. 5b. Crucially, this agreement includes the particular shape of the suppression of the pinch point given by the quadratic photon dispersion around the *Γ*-point, shown in Fig. 6d. In the Supplementary Material we show further evidence that the form of the suppression indeed precisely matches the field theory expectation ${{\mathcal{S}}}({{\boldsymbol{q}}}) \sim {{{\boldsymbol{q}}}}^{2}$. On the other hand, the agreement becomes worse when long-range order sets in. We also note that the first order confinement transition which we observe in Fig. 4 is compatible and analogous to the first-order confinement transition known from conventional compact U(1) gauge theories in 3 + 1 dimensions^70–72^.

#### Excited sectors

The last section provided strong evidence that the spin-1 spiderweb model hosts an extended rank-2 U(1) QSL phase. However, its regime of stability near the RK point is relatively narrow, and its energetic separation from other sectors is small, see Fig. 3b. Here, we demonstrate that rank-2 U(1) QSLs in the spin-1 spiderweb model exist in much broader ranges: We also find them in generic excited sectors, where their stability region in *μ* is considerably larger and where they appear in 90^∘^ rotation symmetric form. Extending our investigation to excited sectors also has a direct physical motivation. Due to the strong Hilbert space fragmentation, the true ground state is hardly accessible by any conventional (numerical or experimental) annealing process, such that the system would come to a rest in excited sectors. Moreover, synthetic platforms such as Rydberg atom arrays^48–50^ facilitate the initialization of the system in a given classical state that may be evolved within its Hilbert space sector.

We start investigating the sector with the 6 × 6 periodic parent state shown in Fig. 3c. The reason for choosing a 6 × 6 state is to complicate the formation of 4 × 4 ground state order, with the intent to increase the stability region of the spin liquid. Figure 6a shows the full momentum dependence of the spin structure factor. At *μ* = 0, where quantum fluctuations are weakest, we find the system to be highly non-ergodic, leading to relatively large statistical uncertainties, despite considerable computational effort. The structure factor ${{\mathcal{S}}}({{\boldsymbol{q}}})$ displays sharp peaks at the smallest finite resolved momenta ***q*** = (*π*/*L*, *π*/*L*). This is indicative of phase separation effects over large distances and with slow dynamics, which is the system’s response to the frustration caused by the 6 × 6 parent state (further explained in the Supplementary Material). For values $\mu \ge 0.2{J}^{{\prime} }$, ergodicity in this sector is restored and suppressed pinch points without any ordering peaks are observed. In contrast to the ground state sector, these patterns show intact fourfold rotational symmetry. In Fig. 6b, we plot ${{\mathcal{S}}}({{\boldsymbol{q}}})$ along a path of high-symmetry points in the Brillouin zone [illustrated in Fig. 6c], showing excellent agreement again with the best fit to the field theory, evidencing a rank-2 U(1) QSL.

To further substantiate the system’s evolution into a QSL phase, in Fig. 6e, we show the maximum of the spin structure factor as a function of *μ*. While the infinite unit cell in the phase-separated regime prevents conventional approaches to locate the phase boundary, we observe that for $\mu \ge 0.2{J}^{{\prime} }$, the maximum becomes nearly independent of the system size, indicating the absence of long-range order. Furthermore, we assess the quality of the fit to the field theory [with spin structure factor ${{{\mathcal{S}}}}^{{{\rm{FT}}}}({{\boldsymbol{q}}})$] via the relative deviation $\delta {{{\mathcal{S}}}}^{{{\rm{FT}}}}={\sum}_{{{\boldsymbol{q}}}\in {{\rm{EBZ}}}}\frac{| {{\mathcal{S}}}({{\boldsymbol{q}}})-{{{\mathcal{S}}}}^{{{\rm{FT}}}}({{\boldsymbol{q}}})| }{{N}_{{{\rm{sites}}}}}$ in the extended Brillouin zone (EBZ) plotted in Fig. 6f. These results also corroborate a transition into a rank-2 U(1) QSL: For $\mu \ge 0.2{J}^{{\prime} }$, the fit becomes increasingly accurate ($\delta {{{\mathcal{S}}}}^{{{\rm{FT}}}}({{\boldsymbol{q}}}) < 0.002$), and in particular the error decreases monotonically with system size.

Lastly, we examine three completely generic excited sectors obtained by randomly generating fracton-free configurations [see Fig. 3c], which are not associated with any periodic parent state. We refer the reader to ref. ^47^ for details regarding the stochastic sampling of these fracton-free configurations. The perfect agreement of the spin structure factor with the prediction of the field theory is also evident in these sectors, as shown Fig. 6g illustrating line cuts of ${{\mathcal{S}}}({{\boldsymbol{q}}})$ for the three sectors at different $\mu \ge 0.6{J}^{{\prime} }$.

This observation lets us conclude that the presence of an extended rank-2 U(1) QSL phase is a general feature of low-lying excited sectors.

## Discussion

The previous sections have established the spin-1 spiderweb model as a platform to realize an extended gapless fracton QSL phase in its ground state and in excited Hilbert space sectors. In this phase, the spin structure factor obtained with error-controlled GFMC matches the predictions of a quantum rank-2 U(1) field theory with extreme precision. In particular, we directly observe the existence of fourfold pinch points, known signatures of gapless fracton phases^40,42,43,73^, and their suppression away from the Rokhsar-Kivelson point. As predicted by our field theory, we find this suppression to conform to the quadratic dispersion of the emergent photon down to small momenta, where it becomes gapless.

The numerically confirmed validity of the effective field theory in Eq. (9) indicates that the conditions under which it is constructed [see discussion below Eq. (9)] are fulfilled in the spin-1 case. Indeed, the assumption of small *B*_□_ fluctuations is known to be non-trivial in two spatial dimensions since phase-slip events *B*_□_ → *B*_□_ + 2*π* in 2+1 dimensional spacetime, so-called *instantons*^46^, can proliferate, gap out the photons and drive a system into an ordered phase (as is the case for the spin-1/2 spiderweb model^47^). Our spin-1 model, however, shows no indications of such phenomena. Although an extremely small photon gap and weak order can never be fully excluded—as is the case with any numerical investigation of finite-size systems—small but finite temperatures could overcome such effects and still realize an effective rank-2 U(1) QSL. The reason why our model apparently evades the instanton effect can possibly be traced back to the absence of Lorentz invariance. As a consequence, possible instanton configurations are highly anisotropic in 2 + 1-dimensional spacetime and might have the shape of world lines with actual particle-like properties (usually called visons) rather than point defects. While an in-depth discussion of such effects is beyond the scope of this work, it is important to note that the field theory is agnostic to the Hilbert space fragmentation of the spiderweb model from which it is derived. This enables the realization of a rank-2 U(1) gauge structure in a multitude of sectors, as we have demonstrated numerically.

From an experimental perspective, Rydberg atom arrays provide a particularly promising platform for realizing our model. In contrast to solid-state implementations, these systems offer a high degree of control over geometry and interactions, and square-lattice arrangements as required for the spiderweb model are routinely realized^50,74,75^. In fact, Rydberg atom arrays have already gained attention for simulations of other lattice gauge theories, such as ${{\mathbb{Z}}}_{2}$ spin liquids^76^. In our case, it is sufficient to directly engineer only the classical Ising part ${{{\mathcal{H}}}}_{1}$: once the associated higher-rank Gauss law constraint is implemented, weak quantum fluctuations (e.g., via laser-induced transverse fields) will generically generate the dynamical terms contained in ${{{\mathcal{H}}}}_{2}$ perturbatively.

The main challenge lies in engineering the required pattern of interaction strengths beyond the natural van-der-Waals form  ~ 1/*r*^6^. However, several recent proposals demonstrate that substantial tunability of effective interactions can be achieved. In particular, Floquet schemes based on periodic modulation of local Rydberg states^77^ and multicolor Rydberg dressing protocols^78^ allow for programmable interaction profiles that can, in principle, approximate the coupling structure required in ${{{\mathcal{H}}}}_{1}$. Another important ingredient is the realization of effective spin-1 degrees of freedom. This can be achieved using multi-level encoding in Rydberg systems using multiple Rydberg states^79–81^. We also note that our finding of fracton spin liquids in excited sectors will aid experiments, which may be prohibited from reaching the true ground state due to Hilbert space fragmentation and glassy dynamics, which are generic features of fracton models.

## Methods

### Green function Monte Carlo

Quantum Monte Carlo methods are numerically exact ways to determine ground state properties of so-called *stochastic Hamiltonians*. Here, we discuss our implementation of the Green function Monte Carlo (GFMC) approach as detailed in ref. ^64^, which we find particularly useful for our given problem, see also refs. ^65,82,83^. In essence, GFMC avoids exhaustive computations in the entire Hilbert space in favor of a random walk, i.e., a Markov chain, to facilitate statistical sampling of observables. The random walk is utilized to realize a projection approach, i.e., projecting out excited states from a trial state $\left\vert {\psi }_{{{\rm{T}}}}\right\rangle$ with finite overlap to the ground state $\left\vert \psi \right\rangle$. One such projection is given in terms of the Hamiltonian ${{\mathcal{H}}}$ as ${(\Lambda -{{\mathcal{H}}})}^{{{\mathcal{P}}}}\left\vert {\psi }_{{{\rm{T}}}}\right\rangle \to \left\vert \psi \right\rangle$, where *Λ* > 0 is a constant and ${{\mathcal{P}}}\to \infty$ the projection order. If ${{\mathcal{G}}}\equiv \Lambda -{{\mathcal{H}}}$ has only non-negative elements, it can be expressed through a Markovian matrix, whose elements define normalized probabilities of transitioning from one classical state to another, allowing the usage of a Monte Carlo approach. While the constant *Λ* can enforce positivity of the diagonal elements, the so-called *sign problem* arises if the Hamiltonian has positive off-diagonal elements. In the present work, we employ the continuous time-limit modification of the method presented in ref. ^84^. This approach performs the exact limit *Λ* → *∞* in which case the projector is exactly equal to an imaginary time evolution operator ${{{\rm{e}}}}^{-{{\mathcal{H}}}\Delta \tau }$, where *Δτ* is a chosen time-step. Note that on a lattice, no Trotterization error occurs regardless of the size of *Δτ*. The requirement $\langle \psi | {\psi }_{{{\rm{T}}}}\rangle={\sum}_{x}\langle \psi | x\rangle \langle x| {\psi }_{{{\rm{T}}}}\rangle \ne 0$ can be seen to be satisfied by choosing *ψ*_T_(*x*) ≡ 〈*x*∣*ψ*_T_〉 > 0 for all configurations $\left\vert x\right\rangle=\vert {S}_{1}^{z},{S}_{2}^{z},\ldots,{S}_{{N}_{{{\rm{sites}}}}}^{z}\rangle$.

#### Importance sampling

Importance sampling is implemented through the trial function itself, which is therefore also referred to as the *guiding wavefunction*. Starting from a spin configuration $\left\vert x\right\rangle=\vert {S}_{1}^{z},{S}_{2}^{z},\ldots,{S}_{{N}_{{{\rm{sites}}}}}^{z}\rangle$, each Markov step consists of sampling a new configuration with a probability proportional to the weight $\langle x| {{\mathcal{G}}}| x^{\prime} \rangle \frac{{\psi }_{{{\rm{T}}}}(x^{\prime} )}{{\psi }_{{{\rm{T}}}}(x)}$. This new configuration then contributes to averages of observables via an accumulation ${\prod }_{i=n}^{n+{{\mathcal{P}}}}{w}_{{x}_{i}}$ of the total weight ${w}_{x}=\mathop{\sum}_{x^{\prime} }\langle x| {{\mathcal{G}}}| x^{\prime} \rangle \frac{{\psi }_{{{\rm{T}}}}(x^{\prime} )}{{\psi }_{{{\rm{T}}}}(x)}$ over the previous steps. This summation can be evaluated efficiently for local Hamiltonians where the number of elements ${{{\mathcal{H}}}}_{xx^{\prime} }=\langle x| {{\mathcal{H}}}| x^{\prime} \rangle$ scales linearly in system size for a given $\left\vert x\right\rangle$. The configurations drawn from the Markov chain this way will be distributed according to an equilibrium condition that is determined by ${{\mathcal{G}}}$ and *ψ*_T_(*x*).

Importantly, if $\left\vert {\psi }_{{{\rm{T}}}}\right\rangle$ is equal to the exact ground state, the variance of the energy is zero and the projection converges at zeroth order. On the other hand, observables which do not commute with the Hamiltonian have a finite variance even if the exact ground state is used, although this variance will generally be larger for less precise wave functions.

While the exact wavefunction is only known at the RK-point, fluctuations and rate of projection convergence are still greatly improved if the guiding wavefunction is reasonably close to the ground state. Here, we choose a standard Jastrow ansatz for the guiding wave function, which captures two-body correlations up to arbitrary length

13 $$\log {\psi }_{{{\rm{T}}}}(x)={\sum}_{i}{m}_{i}{x}_{i}+\frac{1}{2}{\sum}_{ij}{V}_{ij}{x}_{i}{x}_{j},$$

where *m*_*i*_ and *V*_*ij*_ = *V*_*ji*_ are real variational parameters that are optimized using the stochastic reconfiguration method^65^ before the start of the GFMC simulation.

Clearly, the Jastrow ansatz captures the exact RK- wavefunction *ψ*_RK_(*x*) = 1, and is efficient to evaluate numerically. During the Markov chain we only need to evaluate the ratio of wavefunction amplitudes between two configurations that differ by a single cluster flip, i.e. $\left\vert x^{\prime} \right\rangle={{{\mathcal{F}}}}_{\square }\left\vert x\right\rangle$, yielding

14 $$\log \left(\frac{{\psi }_{{{\rm{T}}}}(x^{\prime} )}{{\psi }_{{{\rm{T}}}}(x)}\right)={\sum}_{i}{m}_{i}(x^{{\prime} }_{i}-{x}_{i})+\frac{1}{2}{\sum}_{ij}{V}_{ij}(x^{{\prime} }_{i}x^{{\prime} }_{j}-{x}_{i}{x}_{j}).$$

A single cluster flip only affects eight sites, which we label by □_*k*_ ≡ □_1_, …, □_8_. Using *V*_*ij*_ = *V*_*ji*_, denoting the change in the spin configuration as *F*_*k*_ ∈ ± 1 and further defining the effective local field ${h}_{i}={m}_{i}+{\sum}_{j}{V}_{ji}{x}_{j}$, we may express this ratio as

15 $$\log \left(\frac{{\psi }_{{{\rm{T}}}}(x^{\prime} )}{{\psi }_{{{\rm{T}}}}(x)}\right)={\sum }_{k=1}^{8}{h}_{{\square }_{k}}{F}_{k}+\frac{1}{2}{\sum}_{kk^{\prime} }{V}_{{\square }_{k}{\square }_{k^{\prime} }}{F}_{k}{F}_{k^{\prime} }$$

which may be computed in ${{\mathcal{O}}}(1)$ complexity, independent of the system size (While *h* contains a sum over all sites, it only needs to be updated after each move as ${h}_{j}\to {h}_{j}+{\sum}_{k}{V}_{{i}_{k},j}{F}_{k}$, while the ratio of wavefunctions needs to be evaluated ~*L* × *L* times before each move.).

Due to this efficient evaluation, we found the Jastrow function to outperform more expressive wavefunction approaches (which can describe a larger manifold of wavefunctions) such as restricted Boltzmann machines^85,86^.

#### Many walker formalism

To reduce statistical fluctuations, particularly at large projection times *τ*, we employ the many walker formalism as introduced in ref. ^64^. This approach propagates a population of walkers independently of each other for a few steps *n*_branch_, (or, a small imaginary time *Δτ* ~ 0.1), during which they accumulate their weight as $\mathop{\prod }_{n=1}^{{n}_{{{\rm{branch}}}}}{w}_{{x}_{n}}$ after which they may recombine, ensuring each walker’s survival with probability proportional to their accumulated weight. A larger number of walkers will thus explore the Hilbert space more efficiently, leading to a lower variance for larger projection times as well as a more rapid convergence of the projection scheme.

We note that, unlike other walker population control mechanisms ^83,87^ or in *diffusion Monte Carlo*, no systematic bias is introduced in this approach, regardless of the number of walkers^65^.

#### Ergodicity

While error-controlled, GFMC, as any Markov chain method, may suffer from poor ergodicity. A good diagnostic tool is to compute errors using the standard deviation of several fully independent runs, i.e., initializing the walkers with randomized configurations. For problems with poor ergodicity, the error obtained this way can be significant, as visible in the leftmost panel of Fig. 6b. In the present case, uniformly sampling from a single sector of the fragmented Hilbert space is not possible. Instead, we first initialize *N*_*w*_ ~ 20,000 walkers in a given sector by specifying the initial configuration and subsequently perform a long series of  ≳ 10^7^ fully random cluster flips. This leads to a state of the walker ensemble, which is uncorrelated with the initial configuration.

We emphasize that this procedure does not improve the ergodicity of the Markov chain, but rather serves as a diagnostic tool to provide accurate error estimates.

## Supplementary information

Supplementary info

Transparent Peer Review file

## Data Availability

Code used to perform the numerical simulations presented in this work is openly accessible in the GitHub repository https://github.com/NilsNiggemann/SpiderWebModel.jl, see ref. ^88^.

## Data availability

Simulation data are made available within a Zenodo repository^89^.
