# Supplementary material for: Gapless fracton quantum spin liquid and emergent photons in a 2D spin-1 model
Source: Nat Commun. 2026 Jul 11;17:6131. doi: 10.1038/s41467-026-74797-0 (PMC13365202; doi:10.1038/s41467-026-74797-0)
Supplement: Supplementary file 1 — Supplementary info [file 41467_2026_74797_MOESM1_ESM.pdf]

Supplementary Material for Gapless fracton quantum spin liquid and emergent photons in a 2D spin-1 model

Nils Niggemann,[1,2,3,*] Meghadeepa Adhikary,[4,5] Yannik Schaden-Thillmann,[2,3] and Johannes Reuther[2,3]

[1] *The Abdus Salam International Center for Theoretical Physics (ICTP), Strada Costiera 11, I-34151 Trieste, Italy*
[2] *Helmholtz-Zentrum Berlin für Materialien und Energie, Hahn-Meitner Platz 1, 14109 Berlin, Germany*
[3] *Dahlem Center for Complex Quantum Systems and Fachbereich Physik, Freie Universität Berlin, 14195 Berlin, Germany*
[4] *Department of Physics and Quantum Centers in Diamond and Emerging Materials (QuCenDiEM) group, Indian Institute of Technology Madras, Chennai 600036, India*
[5] *SISSA, Via Bonomea 265, I-34136 Trieste, Italy*

(Dated: June 11, 2026)

I. DERIVATION OF THE GROUND STATE CONSTRAINT

Here, we briefly summarize the derivation of the ground state constraint $\mathcal{C}_{\boxtimes} = 0$, found in more detail in Ref. [1]. We start with the continuum form of the chargeless rank-2 Gauss law, where $E^{\mu\nu}$ is the traceless, symmetric rank-2 electric field and $\mu, \nu \in \{x, y\}$ such that

$$(\partial_x^2 - \partial_y^2)E^{xx}(\boldsymbol{r}) + 4\partial_x\partial_y E^{xy}(\boldsymbol{r}) = 0 \tag{1}$$

In principle, in a rotation-invariant form of the Gauss law $\partial_\mu\partial_\nu E^{\mu\nu} = 0$ a factor 2 instead of a factor 4 would appear in the second term of Eq. (1). However, this would lead to non-uniform prefactors in the corresponding spin constraint and we therefore prefer to use the constraint in Eq. (1). We begin by discretizing the derivatives over unit vectors $\hat{x}, \hat{y}$, for example $\partial_x^2 E^{xx}(\boldsymbol{r}_i) \to E^{xx}(\boldsymbol{r}_i + \hat{x}) - 2E^{xx}(\boldsymbol{r}_i) + E^{xx}(\boldsymbol{r}_i - \hat{x})$. In discretized form, Eq. (1) then becomes

$$\begin{aligned} 0 = &+E^{xx}(\boldsymbol{r}_i + \hat{x}) + E^{xy}(\boldsymbol{r}_i + \hat{x} + \hat{y}) \\ &-E^{xx}(\boldsymbol{r}_i + \hat{y}) - E^{xy}(\boldsymbol{r}_i + \hat{y} - \hat{x}) \\ &+E^{xx}(\boldsymbol{r}_i - \hat{x}) + E^{xy}(\boldsymbol{r}_i - \hat{y} - \hat{x}) \\ &-E^{xx}(\boldsymbol{r}_i - \hat{y}) - E^{xy}(\boldsymbol{r}_i + \hat{x} - \hat{y}) \end{aligned} \tag{2}$$

In order to map this equation to a spin model, we associate the two inequivalent components with independent and unconstrained spin degrees of freedom, each localized on a different sublattice of a square lattice in a unit cell at position $\boldsymbol{r}_i$.

$$E^{xx}(\boldsymbol{r}_i) = -E^{yy}(\boldsymbol{r}_i) = S^z_{\square}(\boldsymbol{r}_i) \tag{3}$$
$$E^{xy}(\boldsymbol{r}_i) = E^{yx}(\boldsymbol{r}_i) = S^z_{\boxtimes}(\boldsymbol{r}_i) \tag{4}$$

By inserting Eqs. (3) and (4) into Eq. (2), we arrive at the constraint $\mathcal{C}_{\boxtimes} = 0$ introduced in the main text.

This construction guarantees the existence of an emergent classical rank-2 U(1) gauge theory in the Gaussian approximation of unconstrained spin degrees of freedom.

II. NUMBER OF CLASSICAL GROUND STATES

We derive an estimate for the dimension n of the constrained Hilbert space in which $\mathcal{C}_{\boxtimes} = 0$ for all eight-site clusters centered around $\boxtimes$. In particular, for a system with N_{sites} spins we express n as $n = b^{N_{\text{sites}}}$ and calculate the base b.

A simple estimate, following Pauling counting arguments, approximates n as $n \approx 3^{N_{\text{sites}}} k^{N_{\text{sites}}/2}$ with the total number of spin-1 Ising configurations given by $3^{N_{\text{sites}}}$ and defining k as the ratio between the number of all configurations satisfying $\mathcal{C}_{\boxtimes} = 0$ for one cluster $\boxtimes$ and the total number of 3^8 Ising configurations in one cluster. Furthermore, $N_{\text{sites}}/2$ is the number of constraints. This approximation gives $b \approx \sqrt{41/27} = 1.232$.

More rigorously, we may also determine b by systematically calculating upper and lower bounds. For estimating an upper bound of b, we consider systems with periodic boundaries and $N_{\text{sites}} = L \times L$ sites. Specifically, we determine n for $L = 4$, $L = 6$, and $L = 8$ by exhaustively generating all states in the constrained subspace. We note that for $L > 8$ this becomes numerically too difficult due to the large number of states. The obtained values are given in Supplementary Table I together with the base b calculated through $b = n^{1/L^2}$. The base b decreases monotonically in L for $L = 4, 6, 8$, and therefore the value $b = 1.468$ for $L = 8$ can be used as an upper bound.

A lower bound of b is found by determining the exact number of states that are obtained by applying fluctuator moves in the configuration of Supplementary Fig. 1(a). This state has a $\sqrt{10} \times \sqrt{10}$ unit cell indicated by red dashed lines and it has one flippable cluster per unit cell (their centers are marked by red dots). Importantly, these flippable clusters do not overlap such that they can be flipped independently. Furthermore, under the condition that flippable clusters do not overlap, their density (one flippable cluster per 10 sites) is maximal in this state. Since each flippable cluster can be in three different configurations, fluctuator moves starting from Supplementary Fig. 1(a) can generate $3^{N_{\text{sites}}/10}$ different states in the constrained subspace, yielding the lower

* nilsf.niggemann@gmail.com

	Number of states n	$b = n^{1/L^2}$
$L = 4$	6 859	1.737
$L = 6$	5 800 827	1.541
$L = 8$	47 067 992 003	1.468

Supplementary Table. I. Dimension n of the constrained subspace for a system with periodic boundaries and $N_{\text{sites}} = L \times L$ sites, where $L = 4$, $L = 6$, and $L = 8$. The third column shows the base b defined by $n = b^{N_{\text{sites}}}$.

bound $b = 3^{1/10} = 1.116$. In total, one finds

$$b = 1.292 \pm 0.176, \tag{5}$$

where the error margins range upward (downward) to the upper (lower) bounds, which agrees with the Pauling estimate. We note that in the case of spin-1/2, the Pauling estimate is found to be surprisingly inaccurate; see Ref. [1] for details.

III. HILBERT SPACE FRAGMENTATION

The dynamics generated by the fluctuators $\mathcal{F}_{\square}$ and $\mathcal{F}_{\square}^{\dagger}$ does not cover the full constrained subspace of the spin-1 spiderweb model, but splits it into many dynamically disconnected sectors. This property, referred to as Hilbert space fragmentation, is known from other fracton models [2–8]. Here, we prove the existence of Hilbert space fragmentation for the spiderweb model, i.e., that the number of dynamically disconnected sectors grows exponentially with the number of sites. While we focus on the case of spin-1, we note that this argument holds for any spin, in particular also spin-1/2; see Ref. [1].

For our proof we consider a new type of fluctuator $\mathcal{F}_{\boxplus}$ shown in Supplementary Fig. 1(b). Its action on a 14-site cluster flips 12 of these spins by two units of angular momentum (i.e. locally acts as $S_i^+ S_i^+$ or $S_i^- S_i^-$) but does not change the two center spins [marked with numbers 1 and 2 in Supplementary Fig. 1(b)]. Importantly, $\mathcal{F}_{\boxplus}$ is defined in a way that it annihilates states where the center spins 1 and 2 are not equal or when they are both in $S^z = 0$ configurations. This may be enforced, via the simple projector $\left[1 - \frac{1}{4}\left(S_1^z - S_2^z\right)^2\right] \times \left(S_1^z\right)^2 \left(S_2^z\right)^2$. In the illustrated case, the center spins have $S^z = 1$, however, our arguments also apply to the case where they are in $S^z = -1$ configurations.

If one only considers the net changes ΔS_i^z at these 14 sites, the fluctuator $\mathcal{F}_{\boxplus}$ is identical to $\mathcal{F}_{\square}\mathcal{F}_{\square}\mathcal{F}_{\square'}\mathcal{F}_{\square'}$ acting on two eight-site clusters centered around the sites $\square$ and $\square'$ ($\square$ and $\square'$ are those sites that both have the sites 1 and 2 as their nearest neighbors). Therefore, $\mathcal{F}_{\boxplus}$ and $\mathcal{C}_{\boxtimes}$ commute for all $\boxtimes$ which means that $\mathcal{F}_{\boxplus}$ operates *within* the constrained subspace. However, if one now considers the consecutive execution of the four operators $\mathcal{F}_{\square}$, $\mathcal{F}_{\square}$, $\mathcal{F}_{\square'}$, $\mathcal{F}_{\square'}$ one finds that there is no order in which these operators can be applied such that they act like $\mathcal{F}_{\boxplus}$,

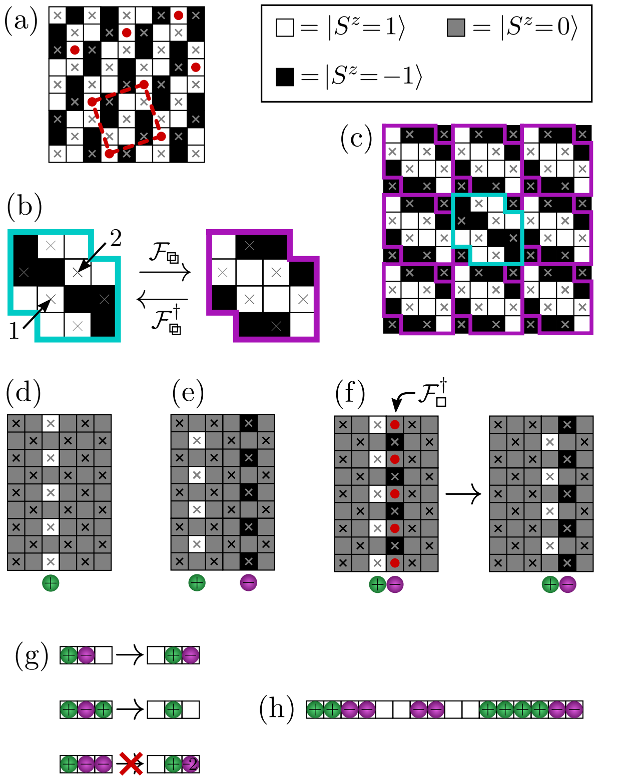

Supplementary Fig. 1. (a) Spin configuration with the largest density of non-overlapping flippable eight-site clusters, to estimate a lower bound for the dimension of the constrained subspace. (b) Definition of the double fluctuator $\mathcal{F}_{\boxplus}$. Note that the two sites 1 and 2 are not flipped by $\mathcal{F}_{\boxplus}$. (c) 4×4 periodic state where the action of $\mathcal{F}_{\boxplus}^{\dagger}$ in any unit cell leads to a new Hilbert space sector. (d) Single S^z-string charge. (e) Single S^z-string dipole. (f) Elementary string-dipole move generated by applying the fluctuator $\mathcal{F}_{\square}^{\dagger}$ on the eight-site clusters around the red dots. (g) Rules for string-dipole moves. While the top and middle moves are allowed, the process at the bottom is forbidden due to the spin-1 constraint $|S_i^z| \leq 1$. (h) Example of a sequence of paired string charges for proving the existence of subextensively many Hilbert space sectors from string states in the spin-1 system.

since they always annihilate the state in Supplementary Fig. 1(b). This is due to the condition that the two center spins 1 and 2 fulfill $S_1^z \neq 0$, $S_2^z \neq 0$ and $S_1^z = S_2^z$. In other words, the action of the double fluctuator $\mathcal{F}_{\boxplus}$ on these 14 sites cannot be expressed as the sequential action of $\mathcal{F}_{\square}$ and $\mathcal{F}_{\square'}$. Consequently, the two 14-site states in Supplementary Fig. 1(b) are in different Hilbert space sectors under the action of $\mathcal{F}_{\square}$ (and $\mathcal{F}_{\square}^{\dagger}$).

The property that $\mathcal{F}_{\boxplus}$ creates new Hilbert space sectors often no longer holds if the motif in Supplementary Fig. 1(b) is part of a larger system with more than 14 sites. This is because in a larger system it may be possible to express $\mathcal{F}_{\boxplus}$ as

$$\mathcal{F}_{\boxplus} = \cdots \mathcal{F}_{\square} \cdots \mathcal{F}_{\square} \cdots \mathcal{F}_{\square'} \cdots \mathcal{F}_{\square'} \cdots, \tag{6}$$

where "$\cdots$" denotes fluctuator moves $\mathcal{F}$ on other sur-

rounding eight-site clusters. To prove the exponential scaling of the number of Hilbert space sectors, it is, however, sufficient to find *one* periodic state with the motif in Supplementary Fig. 1(b), in which $\mathcal{F}_{\boxplus}$ cannot be expressed via elementary fluctuators $\mathcal{F}_{\square}$ as in Eq. (6). Such a configuration is shown in Supplementary Fig. 1(c), which has a 4×4 unit cell containing the motif in Supplementary Fig. 1(b). Another configuration with this property is discussed in Ref. [1]. Note that the action of the double fluctuator $\mathcal{F}_{\boxplus}^{\dagger}$ is illustrated in the center 4×4 unit cell of Supplementary Fig. 1(c). Importantly, none of the states obtained by applying $\mathcal{F}_{\boxplus}^{(\dagger)}$ in different unit cells have eight-site clusters that can be flipped by $\mathcal{F}_{\square}$ or $\mathcal{F}_{\square}^{\dagger}$. Consequently, the action of $\mathcal{F}_{\boxplus}^{\dagger}$ in any 4×4 unit cell leads to a new Hilbert space sector. A lower bound for the number of sectors can now be estimated as $2^{N_{\mathrm{sites}}/16}$, demonstrating its exponential scaling with system size. However, note that each of these sectors is trivial in the sense that it contains only a single state.

We emphasize that in a generic spin-1 configuration with the 14-site motif in Supplementary Fig. 1(b), the action of $\mathcal{F}_{\boxplus}$ will usually *not* result in a new sector because $\mathcal{F}_{\boxplus}$ can be expressed as in Eq. (6). In rare exceptions [Supplementary Fig. 1(c)], the creation of new Hilbert space sectors via $\mathcal{F}_{\boxplus}$ is only possible because the 14-site motif is trapped in an environment with inactive or severely limited $\mathcal{F}_{\square}$-dynamics (e.g. due to the absence of local $S_i^z = 0$ states). Stated differently, we could not find a spin-1 state where the application of $\mathcal{F}_{\boxplus}$ creates a new sector while the $\mathcal{F}_{\square}$-dynamics in these sectors is connected and can spread over the whole system. We could generally not identify any mechanism to construct exponentially many Hilbert space sectors that have non-trivial dynamics. We therefore conclude that while spin states like in Supplementary Fig. 1(c) can be used to prove the existence of exponentially many sectors, they are highly artificial and the total number of states they contain covers only a small part of the constrained Hilbert space.

On the other hand, there exists a way to construct subextensively many sectors (i.e., whose number scales exponentially with the linear system size L) that have non-trivial and collective dynamics, possibly hosting a fracton spin liquid. Their construction is based on the string-like configuration on sublattice 1 shown in Supplementary Fig. 1(d). In the following, we define the operator $\mathcal{G}_{|} = \prod S_{\boxtimes}^{+}$, which creates such a string when acting on a homogeneous $S_i^z = 0$ state. We assign a 'string-charge' $\mathcal{Q} = \pm 1$ to these states, indicating whether the strings are built from $S_i^z = 1$ or from $S_i^z = -1$ states. We call such configurations 'S^z-strings' and mark them with green balls (for $\mathcal{Q} = +1$) or violet balls (for $\mathcal{Q} = -1$) in Supplementary Fig. 1(d)-(h). The term 'charge' in this context should not be confused with the fractonic matter charges ρ. All configurations with one or more strings are translation-invariant in the y direction and fulfill all constraints $\mathcal{C}_{\boxtimes} = 0$. The following discussion considers only such translation-invariant states and treats them as representative states for the entire Hilbert space sectors they belong to, in which y-translation invariance of individual states is typically broken. In a similar way, our arguments also hold for horizontal or diagonal strings (note, however, that a diagonal string has to be defined on sublattice 2 in order to respect all ground-state constraints).

The operator $\mathcal{G}_{|}$ does not commute with the conserved subdimensional magnetizations $M_{\diagdown}$, $M_{\diagup}$ in Fig. 2 of the main text and thus cannot be constructed by repeated applications of $\mathcal{F}_{\square}$. Therefore, adding a S^z-string generates a new Hilbert space sector. Next, we consider a string-dipole consisting of a $\mathcal{Q} = +1$ string and a $\mathcal{Q} = -1$ string, as shown in Supplementary Fig. 1(e), which is created by the operator $\mathcal{G}_{|_1} \mathcal{G}_{|_2}^{\dagger}$. Since such operators also cannot be built from individual fluctuators $\mathcal{F}_{\square}$, because this would require *fractional* fluctuator moves (see discussion in Fig. 2 of Ref. [1]), adding a string-dipole again gives rise to a new Hilbert space sector. The simplest change of one string configuration into another, which does *not* create a new Hilbert space sector, is the translation of a string-dipole in the perpendicular direction as shown in Supplementary Fig. 1(f). This string-dipole move can be generated by applying fluctuators $\mathcal{F}_{\square}^{\dagger}$ on the eight-site clusters around the red dots in Supplementary Fig. 1(f) and corresponds to the insertion of a string-quadrupole.

Importantly, however, this motion of string dipoles is restricted by the spin constraint $S_i^z \in \{-1, 0, 1\}$. To see this, we summarize the rules for string moves in Supplementary Fig. 1(g), where we only show the effective one-dimensional system of green/violet string charges along the x direction and omit the y direction. The move illustrated in the top panel of Supplementary Fig. 1(g) is just the elementary dipole translation of Supplementary Fig. 1(f). The middle panel of Supplementary Fig. 1(g) also shows an allowed elementary dipole move where the '$+-$' dipole in the sequence '$+--+$' is moved to the right by one lattice spacing, involving the annihilation of a positive and negative string-charge. On the other hand, as shown in the bottom panel of Supplementary Fig. 1(g), a '$+-$' dipole cannot be moved to the right if it is blocked by another '$-$' string charge, as this would generate sites with $S_i^z = -2$. Therefore, a string dipole cannot be moved across a single string-charge by fluctuator moves $\mathcal{F}_{\square}$.

This blocking effect in the motion of string-dipoles due to the spin length constraint gives rise to a subextensive scaling of the number of Hilbert space sectors. To understand this and to estimate a lower bound for the number of sectors, we consider a *subset* of all possible string configurations in which all arrangements of string charges ($\mathcal{Q} = -1$, $\mathcal{Q} = 0$ or $\mathcal{Q} = +1$) occur in nearest-neighbor pairs of equal charges [see Supplementary Fig. 1(h) for an example]. Importantly, dipole motion is completely blocked in all of these states due to the forbidden process in the bottom panel of Supplementary Fig. 1(g), such that different states cannot be transformed into each other by $\mathcal{F}_{\square}$ moves. Therefore, each of these paired string configurations can be regarded as a representative state

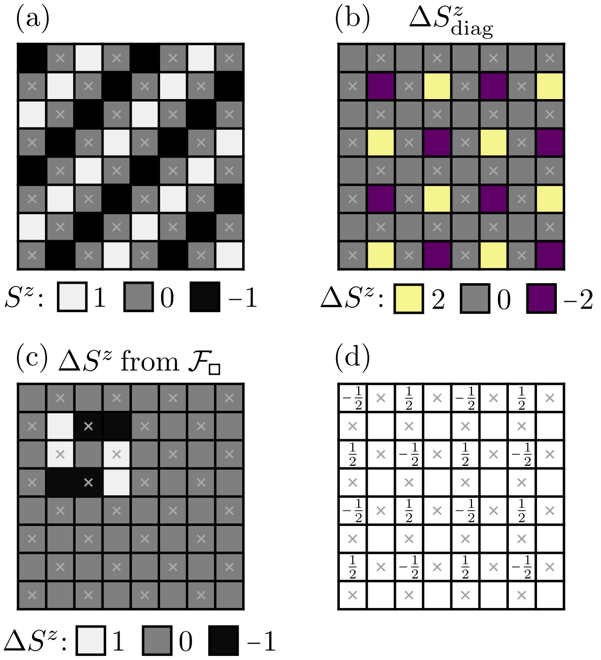

Supplementary Fig. 2. (a) 90° rotated version of the diagonal stripe state in Fig. 3(a) of the main text. (b) Difference $\Delta S^z_{\mathrm{diag}}$ between the diagonal stripe states in Fig. 3(a) of the main text and in (a). (c) Spin changes ΔS^z from the application of $\mathcal{F}_{\square}$ in one eight-site cluster. (d) Multiplicities of the applications of $\mathcal{F}_{\square}$ to generate $\Delta S^z_{\mathrm{diag}}$ in (b).

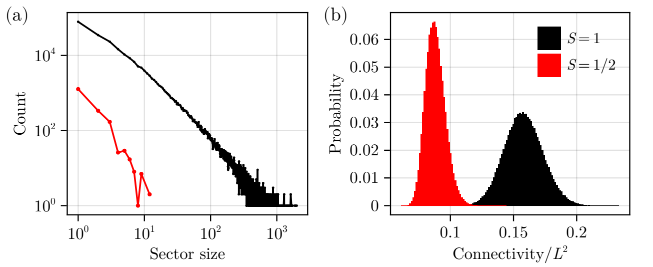

Supplementary Fig. 3. (a) Histogram of connected-sector sizes for spin-1/2 and spin-1 for $L = 6$ obtained via exact enumeration of solutions. (b) Probability distribution of connectivity of configurations in the staircase (spin-1/2) and diagonal stripe (spin-1) sector for $L = 28$, obtained via GFMC sampling of configurations at the RK point.

in distinct Hilbert space sectors. The number of these configurations scales subextensively as $3^{L_x/2}$ where the base 3 stands for the three possible charges $\mathcal{Q} = -1$, $\mathcal{Q} = 0$ or $\mathcal{Q} = +1$ and the factor 1/2 in the exponent is due to the pairing of strings.

Crucially, in contrast to the extensively many sectors constructed via the double moves $\mathcal{F}_{\boxplus}$, the subextensively many sectors from string configurations usually allow for non-trivial, collective dynamics with overlapping fluctuator moves $\mathcal{F}_{\square}$. We expect that all states in the subextensively many string sectors altogether cover a much larger part of the constrained Hilbert space than the sectors from $\mathcal{F}_{\boxplus}$-moves.

IV. RELATION BETWEEN FRAGMENTATION AND SPIN QUANTUM NUMBER

In Ref. [1], it was found that fragmentation has prohibitive effects on the dynamics of the spin-1/2 spiderweb model. In the main text, the effects of fragmentation were found to be qualitatively weaker for spin-1. To quantify this difference, we compare the number and sizes of sectors for spin-1/2 and spin-1 for different system sizes $L \times L$ with periodic boundaries by exact counting of all possible configurations. The results are summarized in Supplementary Table II. As both the number of fracton-free configurations and sectors grow exponentially in L^2, it is useful to define the fragmentation coefficient

$$f = (n_{\mathrm{Sectors}}/n_{\mathrm{Total}})^{1/L^2}, \tag{7}$$

where n_{Total} is the total number of fracton-free spin states and n_{Sectors} is the number of Hilbert space sectors for a given system size L. If there is no fragmentation, the number of sectors grows subexponentially as $L \to \infty$, whereas $n_{\mathrm{Total}} = b^{L^2}$ and thus $f = 0$. On the other hand, for maximal fragmentation each configuration forms its own sector, $n_{\mathrm{Sectors}} = n_{\mathrm{Total}}$ and thus $f = 1$. In the case of the spiderweb model, we find $f \sim 0.933$ for spin-1 and $f = 0.982$ for spin-1/2. While both are indicative of very strong fragmentation, the value for spin-1 is significantly smaller, consistent with the observation of weaker fragmentation. While f is a useful measure for the degree of fragmentation, it does not capture the effect that fragmentation has on the dynamics within individual sectors. To quantify this, we compare the sizes of sectors in Fig. 3(a), and find that for spin-1, the size of Hilbert space sectors increases considerably compared to spin-1/2 at an equal system size of $L = 6$. Another useful measure is the connectivity which specifies how many states can be reached from a starting configuration by applying a single eight-site fluctuator move. In Fig. 3(b) we display the considerably larger average connectivity of configurations for spin-1 in the diagonal stripe sector as compared to the lowest energy sector reported in Ref. [1] for spin-1/2 (dubbed staircase sector). Both results, respectively, were obtained by GFMC sampling of configurations at the RK point for $L = 28$.

L	Total		Sectors		f	
S	1/2	1	1/2	1	1/2	1
4	216	6861	184	2621	0.99	0.942
6	5912	5800829	3732	473717	0.987	0.933
8	350872	47067992003	108534	—	0.982	—
10	37403668	—	6042418	—	0.982	—
12	10103561614	—	—	—	—	—

Supplementary Table. II. Number of fracton-free states, disconnected sectors and fragmentation coefficient from Eq. (7) for spin-1/2 and spin-1 on $L \times L$ lattices. Dashes indicate data that was infeasible to obtain due to memory requirements.

V. DYNAMICALLY DISCONNECTED DIAGONAL STRIPE SECTORS

Here we show that 90° rotated versions of the diagonal stripe state lie in dynamically disconnected sectors under the action of $\mathcal{F}_{\square}$ and $\mathcal{F}_{\square}^{\dagger}$. This property explains the broken 90° rotation symmetry of the spin structure factor in Fig. 5(a) of the main text and shows that this asymmetry is not a consequence of spontaneous lattice symmetry breaking, but rather due to the asymmetry of the diagonal stripe state itself.

In Supplementary Fig. 2(a) we show a 90° rotated version of the diagonal stripe state in Fig. 3(a) of the main text. For the system to tunnel between both states, the z-components of the spins have to change by the amount illustrated in Supplementary Fig. 2(b), where $\Delta S^z_{\mathrm{diag}}$ is the difference between the diagonal stripe state in Fig. 3(a) of the main text and the one in Supplementary Fig. 2(a). In a tunneling process this difference has to be generated by repeated actions of $\mathcal{F}_{\square}$ (or $\mathcal{F}_{\square}^{\dagger}$) on the different eight-site clusters $\square$. As an example, the change ΔS^z from $\mathcal{F}_{\square}$ for one particular eight-site cluster $\square$ is illustrated in Supplementary Fig. 2(c). The problem of describing this tunneling process with $\mathcal{F}_{\square}$ can be considered as a linear algebra problem that consists of finding a linear combination of the eight-site changes ΔS^z in Supplementary Fig. 2(c) that yields $\Delta S^z_{\mathrm{diag}}$ in Supplementary Fig. 2(b). It needs to be taken into account that the $N_{\mathrm{sites}}/2$ fluctuators $\mathcal{F}_{\square}$ on all clusters $\square$ are not linearly independent but have rank $N_{\mathrm{sites}}/2 - 1$, as discussed in detail in Sec. II.B in Ref. [1]. Therefore, to find a unique solution, one of the fluctuators $\mathcal{F}_{\square}$ needs to be omitted to ensure that the other fluctuators $\mathcal{F}_{\square}$ form a set of basis vectors for the local moves in the constrained subspace. Here, without loss of generality, we have omitted the fluctuator in row 1, column 2 (when counted from the bottom left corner) of the lattice array in Supplementary Fig. 2. The solution to express $\Delta S^z_{\mathrm{diag}}$ via local fluctuators, obtained by simple matrix inversion, is illustrated in Supplementary Fig. 2(d) where the numbers a on the sublattice 2 sites $\square$ correspond to the multiplicities $(\mathcal{F}_{\square})^a$ with which the integer spin changes ΔS^z in the eight-site clusters have to be applied. Importantly, these multiplicities contain *non-integer* $a = \pm 1/2$ values, showing that fractional applications of $\mathcal{F}_{\square}$ would be needed to tunnel between both states. In other words, no sequential applications of $\mathcal{F}_{\square}$ exist that can realize this process and, consequently, both diagonal stripe states lie in different Hilbert space sectors. This result is independent of the choice of fluctuator $\mathcal{F}_{\square}$ that is omitted in the set of basis vectors.

We note that in addition to 90° rotated versions of diagonal stripe states, also time-reversed ($S^z \to -S^z$) versions exist. However, time-reversed partners of diagonal stripe states are found to be in the *same* Hilbert space sector, which implies that the diagonal stripe order at $\mu < 0.8J'$ in Fig. 4 of the main text is associated with spontaneous time-reversal symmetry breaking.

VI. MAXIMALLY FLIPPABLE STATES IN THE 6×6 SECTOR

The observation of broken ergodicity at low μ in the sector of the 6×6 state in Fig. 3(c) of the main text is related to the system's inability to exhibit 4×4 order: While the 6×6 state has a large number of flippable clusters, numerical results indicate that other, more complicated, configurations exist in this sector with an even larger number. These configurations, such as the one shown in Fig. 4(a), and configurations related by lattice symmetries, are separated by large energetic barriers for small μ, leading to exceedingly large autocorrelation times. At larger values of μ, energetic barriers are lowered, while fluctuations between larger networks of spin configurations become favorable, allowing for ergodic quantum tunneling within the entire sector. In order to find this configuration, we initialized 3840 independent GFMC simulations at $\mu = 0$ with a single walker and recorded each configuration encountered. To confirm that the correct solution was found, we repeated this procedure five times, finding equivalent configurations (up to lattice translations). The result is shown in Fig. 4(a), featuring a phase separation effect with a large

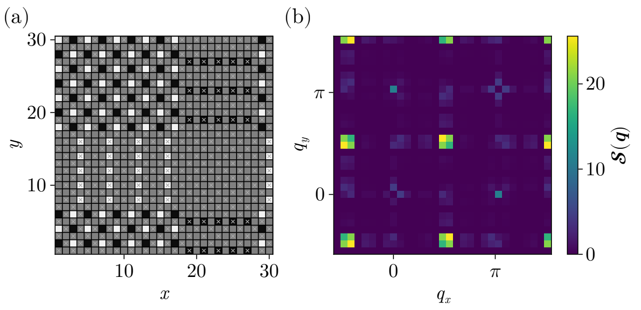

Supplementary Fig. 4. (a) One of the maximally flippable states found in the 6×6 sector and (b) its corresponding spin structure factor $\mathcal{S}(\boldsymbol{q})$.

domain with a 4×4 order. By computing $\mathcal{S}(\boldsymbol{q})$ for this configuration [Fig. 4(b)], we may explain the features at $\boldsymbol{q} = (\pi/L, \pi/L)$ and symmetry-related points in Fig. 6(a) of the main text, as a result of the large size of the magnetic domains. On the other hand, this configuration still shows a strong peak at $\boldsymbol{q} = (\pi, \pi)$ which is however difficult to resolve in Fig. 6(a) of the main text due to the considerable uncertainties resulting from the broken ergodicity.

VII. DETAILS ON THE EFFECTIVE RANK-2 U(1) FIELD THEORY

Our Maxwellian field theory in Eq. (9) of the main text is derived by expressing the spin flip operators in $\mathcal{H}_2$ in terms of rotor variables $S_i^{\pm} = \sqrt{2}e^{\pm\imath A_i^{\alpha}}$ where A_i^{α} takes the role of a generalized 'vector' potential. The operators A_i^{α} (which follow the convention that $\alpha = xy$ when i is on sublattice 1 and $\alpha = xx$ when i is on sublattice 2) are the components of a trace-free and symmetric matrix-valued field, i.e., $A_i^{xx} = -A_i^{yy}$ and $A_i^{xy} = A_i^{yx}$. Furthermore, A_i^{α} is compact, i.e., its eigenvalues lie in the interval $[0, 2\pi]$. The z-components of the spins S_i^z take the role of a conjugate integer-valued matrix electric field $S_i^z = E_i^{\alpha}$ with $[A_i^{\alpha}, E_i^{\alpha}] = \imath$. Note that when i is on sublattice 1 (2), we also use the notation $A_i^{xy} \equiv A_{\boxtimes}^{xy}$ ($A_i^{xx} \equiv A_{\square}^{xx}$) and equivalently for E_i^{α}. With these definitions $\mathcal{H}_2$ has the form

$$\mathcal{H}_2 \sim -\sum_{\square} \cos B_{\square} \sim +\sum_{\square} B_{\square}^2 \tag{8}$$

where $B_{\square}$ is defined for each cluster $\square$ as given in Eq. (8) of the main text and the rightmost expression in Eq. (18) of the main text represents the expansion of $\mathcal{H}_2$ in lowest non-trivial order in $B_{\square}$. Furthermore, in order to suppress electric field states $E_i^{\alpha} \notin \{-1, 0, 1\}$, a term $\sim \sum_i (E_i^{\alpha})^2$ in the field theory is required. Together with the RK potential $\sim \sum_{\square} \mathcal{N}_{\square}^2$ this yields the effective field theory in Eq. (9) of the main text. An important property of this theory that distinguishes it from conventional U(1) gauge theories is the absence of electromagnetic duality. This is already evident from the different properties of the electric and magnetic fields where the former is a matrix while the latter is a one-component object.

Note that our mapping to an effective field theory could, in principle, also be applied to the spin-1/2 spiderweb model. This would require a change of the (irrelevant) prefactor $\sqrt{2}$ in Eq. (6) of the main text and a redefinition of the electric field to ensure that it only assumes integer values. The resulting field theory, known e.g. from spin-1/2 quantum spin ice, is called 'frustrated' [9] due to the lack of a well-defined vacuum of electric fields. On the other hand, our field theory for a spin-1 system has the advantage of a unique vacuum state corresponding to the homogeneous $S_i^z = 0$ configuration.

The field theory has a local gauge freedom that follows from the rank-2 Gauss' law and amounts to the invariance under the operation [10]

Quantities on sublattice 1 (= center of $\boxtimes$ clusters):	Quantities on sublattice 2 (= center of $\square$ clusters):
$\mathcal{C}$ (constraint)	$\mathcal{F}$ (fluctuator)
ρ (fractons)	B (magnetic field)
A^{xy}, E^{xy} (fields of U(1) theory)	A^{xx}, E^{xx} (fields of U(1) theory)
f (gauge transformation)	$\mathcal{N}^2$ (RK potential)

Supplementary Table. III. Definitions of the two sublattices of the spiderweb model and location of different quantities.

$$U(f_{\boxtimes}) = \exp\left(\imath \sum_{\boxtimes} f_{\boxtimes}\mathcal{C}_{\boxtimes}\right) \tag{9}$$

where $f_{\boxtimes}$ is an arbitrary function defined for each cluster $\boxtimes$ or, stated differently, $f_{\boxtimes}$ is located on sublattice 1. To see this, we use

$$\mathcal{C}_{\boxtimes} = E_{\boxtimes_1}^{xx} + E_{\boxtimes_2}^{xy} - E_{\boxtimes_3}^{xx} - E_{\boxtimes_4}^{xy} + E_{\boxtimes_5}^{xx} + E_{\boxtimes_6}^{xy} - E_{\boxtimes_7}^{xx} - E_{\boxtimes_8}^{xy} \tag{10}$$

and rearrange the sum in the exponent of Eq. (9), yielding

$$\begin{aligned} U(f_{\boxtimes}) = &\exp\left[\imath \sum_{\boxtimes} E_{\boxtimes}^{xy}(f_{\boxtimes_2} - f_{\boxtimes_4} + f_{\boxtimes_6} - f_{\boxtimes_8})\right] \times \\ &\exp\left[\imath \sum_{\square} E_{\square}^{xx}(f_{\square_1} - f_{\square_3} + f_{\square_5} - f_{\square_7})\right]. \end{aligned} \tag{11}$$

Here, the labels $\boxtimes_a$ and $\square_a$ use the convention explained below Eq. (2) of the main text where, e.g., $f_{\square_1}$ is a site to the right of the center of a $\square$ cluster, such that $f_{\square_1}$ is still defined on sublattice 1 (center of a $\boxtimes$ cluster). Since $\exp(\imath\theta E_i^{\alpha})$ with $\theta \in \mathbb{R}$ shifts $A_i^{\alpha} \to A_i^{\alpha} + \theta$, the operation in Eq. (11) can be written as

$$\begin{aligned} A_{\boxtimes}^{xy} &\to A_{\boxtimes}^{xy} + f_{\boxtimes_2} - f_{\boxtimes_4} + f_{\boxtimes_6} - f_{\boxtimes_8}, \\ A_{\square}^{xx} &\to A_{\square}^{xx} + f_{\square_1} - f_{\square_3} + f_{\square_5} - f_{\square_7}. \end{aligned} \tag{12}$$

By simple bookkeeping of all terms it can be checked that Eq. (8) of the main text is indeed invariant under this transformation.

In Supplementary Table III we list all the quantities occurring in the spiderweb model and in the effective field theory and we specify the sublattice on which they are defined.

The gauge transformation in Eq. (12) becomes more transparent in a continuum description where it reads

$$\begin{aligned} A^{xy} &\to A^{xy} + 4\partial_x\partial_y f, \\ A^{xx} &\to A^{xx} + (\partial_x^2 - \partial_y^2) f. \end{aligned} \tag{13}$$

Using the continuum definition of the magnetic field in Eq. (8) of the main text,

$$B = -4\partial_x\partial_y A^{xx} + (\partial_x^2 - \partial_y^2) A^{xy}, \tag{14}$$

the invariance of B under the gauge transformation in Eq. (13) is immediately obvious. The special property of Eq. (14) is that B is constructed from *second* derivatives of A^{xx} and A^{xy}. This is in contrast to a three-dimensional scalar charge rank-1 or rank-2 U(1) gauge theory where one derivative is sufficient to construct a gauge invariant magnetic field [11]. The relation between B and $A^{\mu\nu}$ in Eq. (14) also makes the lack of Lorentz-invariance of the effective field theory apparent. To see this, we consider the system's Lagrangian $\mathcal{L}$ which contains a term $\sim (\partial_t A^{\mu\nu})^2$ with a first derivative in time describing the electric field contribution $(E^{\mu\nu})^2$. Furthermore, $\mathcal{L}$ contains a term $\sim B^2$ which, according to Eq. (14), contains second spatial derivatives. This unequal treatment of space and time is incompatible with Lorentz invariance.

Returning to the lattice version of our rank-2 U(1) gauge theory [see Eq. (9) of the main text], this Hamiltonian describes a Gaussian theory whose eigenmodes can be calculated analytically. To this end we rewrite A_i^α and E_i^α in terms of bosonic operators a_i^α, $(a_i^\alpha)^\dagger$,

$$A_i^\alpha = \frac{1}{\sqrt{2}}\left[a_i^\alpha + (a_i^\alpha)^\dagger\right] \ , \quad E_i^\alpha = \frac{1}{\sqrt{2}\imath}\left[a_i^\alpha - (a_i^\alpha)^\dagger\right] \ , \tag{15}$$

which fulfill the standard commutation relation $[a_i^\alpha, (a_i^\alpha)^\dagger] = 1$. Furthermore, we Fourier-transform the bosonic operators using

$$a_i^\alpha = \sqrt{\frac{2}{N_{\text{sites}}}} \sum_{\boldsymbol{q}} e^{\imath \boldsymbol{q}\cdot\boldsymbol{r}_i} a^\alpha(\boldsymbol{q}) \ . \tag{16}$$

Here, $\boldsymbol{r}_i$ is the real-space position of site i and the sum only includes momenta $\boldsymbol{q}$ in the first Brillouin zone. Note that our model consists of two inequivalent sublattices such that the first Brillouin zone can, for example, be chosen as $q_x \in [-\pi/2, \pi/2]$, $q_y \in [-\pi, \pi]$. With these definitions, $\mathcal{H}_{\text{eff}}$ from Eq. (9) of the main text can be written as

$$\mathcal{H}_{\text{eff}} = \sum_{\boldsymbol{q}} A^\dagger(\boldsymbol{q}) H_{\text{eff}} A(\boldsymbol{q}) \ , \tag{17}$$

where $A(\boldsymbol{q})$ is a four-component operator

$$A(\boldsymbol{q}) = \left(a^{xy}(\boldsymbol{q}), a^{xx}(\boldsymbol{q}), [a^{xy}(-\boldsymbol{q})]^\dagger, [a^{xx}(-\boldsymbol{q})]^\dagger\right) \tag{18}$$

and the 4×4 matrix H_{eff} is given by

$$H_{\text{eff}} = K V_+^T V_+ + W V_-^T V_- + \frac{U}{4}\begin{pmatrix} 1 & 0 & -1 & 0 \\ 0 & 1 & 0 & -1 \\ -1 & 0 & 1 & 0 \\ 0 & -1 & 0 & 1 \end{pmatrix} \tag{19}$$

with

$$V_\pm = (c_x - c_y, 2s_x s_y, \pm(c_x - c_y), \pm 2 s_x s_y) \tag{20}$$

and $c_\mu = \cos q_\mu$, $s_\mu = \sin q_\mu$ with $\mu = x, y$.

According to the standard procedure of a Bogoliubov transformation, to solve the model, we need to rewrite $\mathcal{H}_{\text{eff}}$ in terms of Bose operators $C(\boldsymbol{q})$ for the eigenmodes,

$$C(\boldsymbol{q}) = (c_1(\boldsymbol{q}), c_2(\boldsymbol{q}), c_1^\dagger(\boldsymbol{q}), c_2^\dagger(\boldsymbol{q})) \tag{21}$$

where $C(\boldsymbol{q}) = \mathcal{J}(\boldsymbol{q})A(\boldsymbol{q})$ such that $(\mathcal{J}^\dagger)^{-1} H_{\text{eff}} \mathcal{J}^{-1}$ is diagonal. Note that, to preserve the bosonic commutation relations of $C(\boldsymbol{q})$, the 4×4 matrix $\mathcal{J}(\boldsymbol{q})$ needs to be a *paraunitary* transformation that satisfies $\mathcal{J}^\dagger g \mathcal{J} = g$ with $g = \text{diag}(1,1,-1,-1)$ [12]. A subtlety arises because of the gauge invariance of $\mathcal{H}_{\text{eff}}$ which manifests in a bosonic zero mode. Since a paraunitary transformation $\mathcal{J}(\boldsymbol{q})$ is not defined in this case, we introduce an additional term in the Hamiltonian of the form $2d\sum_i (A_i^\alpha)^2$ which breaks gauge invariance and which allows us to explicitly calculate $\mathcal{J}(\boldsymbol{q})$. The gauge-invariant limit $d \to 0$ can be recovered at the end of the calculation. The transformation matrix $\mathcal{J}(\boldsymbol{q})$ is found to be

$$\mathcal{J}(\boldsymbol{q}) = \frac{1}{\sqrt{8(L_1^2 + L_2^2)}} \times \\ \times \begin{pmatrix} L_1\xi_+ & L_2\xi_+ & -L_1\xi_- & -L_2\xi_- \\ -L_2\lambda_+ & L_1\lambda_+ & L_2\lambda_- & -L_1\lambda_- \\ -L_1\xi_- & -L_2\xi_- & L_1\xi_+ & L_2\xi_+ \\ L_2\lambda_- & -L_1\lambda_- & -L_2\lambda_+ & L_1\lambda_+ \end{pmatrix} \tag{22}$$

with

$$\xi_\pm = \left(\frac{U}{d}\right)^{1/4} \pm 2\left(\frac{d}{U}\right)^{1/4} , \tag{23}$$

$$\lambda_\pm = \sqrt{2}\left[\left(\frac{\eta_2}{\eta_1}\right)^{1/4} \pm \left(\frac{\eta_1}{\eta_2}\right)^{1/4}\right] , \tag{24}$$

$$\eta_1 = d + K\left[(c_x - c_y)^2 + 4s_x^2 s_y^2\right] , \tag{25}$$

$$\eta_2 = \frac{U}{4} + W\left[(c_x - c_y)^2 + 4s_x^2 s_y^2\right] , \tag{26}$$

and L_1, L_2 are the components of the *constraint vector* [1], given by

$$L_1(\boldsymbol{q}) = -4s_x s_y \ , \ L_2(\boldsymbol{q}) = 2(c_x - c_y) \ . \tag{27}$$

Diagonalizing H_{eff} with $\mathcal{J}$ leads in the limit $d \to 0$ to a zero mode due to the system's gauge freedom and a single photon mode with the dispersion

$$\begin{aligned} \omega(\boldsymbol{q}) &= 2\sqrt{\eta_1\eta_2} \\ &= 2\sqrt{K\left[(c_x - c_y)^2 + 4s_x^2 s_y^2\right]} \times \\ &\quad \times \sqrt{\frac{U}{4} + W\left[(c_x - c_y)^2 + 4s_x^2 s_y^2\right]} . \end{aligned} \tag{28}$$

This photon dispersion is gapless at $\boldsymbol{q} = (0,0)$ and $\boldsymbol{q} = (\pi,\pi)$. An expansion of $\omega(\boldsymbol{q})$ around these two points yields for $U \neq 0$ in lowest non-vanishing order

$$\omega(\boldsymbol{q}) \approx \sqrt{\frac{KU}{4}}\sqrt{q_x^4 + 14q_x^2 q_y^2 + q_y^4} \ . \tag{29}$$

This function is quadratic in any radial direction away from the gapless points, however, it does not have a continuous rotation symmetry around these points, as discussed in Sec. II.A in Ref. [1]. On the other hand, exactly at the RK-point $U = 0$, the photon dispersion becomes quartic at long wavelengths,

$$\omega(\boldsymbol{q}) \approx \sqrt{\frac{KW}{4}\left(q_x^4 + 14 q_x^2 q_y^2 + q_y^4\right)} \ . \tag{30}$$

Another prediction of the field theory is the spin structure factor

$$\mathcal{S}(\boldsymbol{q}) = \frac{1}{N_{\text{sites}}} \sum_{i,j} \langle S_i^z S_j^z \rangle e^{\imath \boldsymbol{q}\cdot(\boldsymbol{r}_i - \boldsymbol{r}_j)}, \tag{31}$$

which can be obtained by expressing the spin operators S_i^z in terms of a_i^α, $(a_i^\alpha)^\dagger$ bosons [Eq. (7) of the main text and Eq. (15)], transforming them into the eigenbasis of C bosons using the matrix $\mathcal{J}$, and exploiting that the ground state is free of any photon excitations. For $d \to 0$ this yields

$$\begin{aligned}\mathcal{S}(\boldsymbol{q}) &= \sqrt{\frac{\eta_1}{\eta_2}} \frac{(L_1 - L_2)^2}{L_1^2 + L_2^2} \\ &= \frac{\sqrt{K}(c_x - c_y + 2 s_x s_y)^2}{\sqrt{(c_x - c_y)^2 + 4 s_x^2 s_y^2}\sqrt{\frac{U}{4} + W\left[(c_x - c_y)^2 + 4 s_x^2 s_y^2\right]}}.\end{aligned} \tag{32}$$

Note that for fitting this function to numerical results, we need to define only two truly independent fitting parameters (A, r) via $(K, W, U) = (4A^2, 1, r)$, where A only modifies the structure factor by a global scaling, which cannot be fixed by sum rules for spin 1, and r tunes the relative strength of the RK potential. In the RK limit $U \to 0$ where $\eta_1/\eta_2 = K/W$ is a constant, this expression becomes (up to a prefactor) identical to the classical spin structure factor in Gaussian approximation given by $\mathcal{S}_{\text{class}}(\boldsymbol{q}) = \frac{(c_x - c_y + 2 s_x s_y)^2}{(c_x - c_y)^2 + 4 s_x^2 s_y^2}$ [1]. This is expected because at the RK point a ground state can be constructed by an equal weight superposition of all S_i^z basis states in a given Hilbert space sector, similar to a classical (non-coherent) superposition. Furthermore, at $W = 0$ when $\eta_2 = U/4$ is a constant, the spin structure factor in Eq. (32) corresponds to the classical result, multiplied by the photon dispersion $\omega(\boldsymbol{q})$ which suppresses the fourfold pinch points around their center. The interpolation between both limits is determined by the term $\sqrt{U/4 + W\left[(c_x - c_y)^2 + 4 s_x^2 s_y^2\right]}$ in the denominator of Eq. (32). For finite $W > 0$ there is a threshold momentum q_c (which decreases with increasing W) above which the W-term dominates and the spin structure factor resembles the classical one. On the other hand, for $q \lesssim q_c$ the U-term dominates and the spin structure factor is suppressed.

The photon dispersion $\omega(\boldsymbol{q})$ and the spin structure factor $\mathcal{S}(\boldsymbol{q})$ of the 90° rotation symmetry broken field theory with the additional term in Eq. (12) of the main text follow straightforwardly from Eq. (28) and Eq. (32) by the replacement

$$U \to U[1 + 2p(1 + \cos(q_x - q_y))], \tag{33}$$

where $p = U'/U$. As pointed out in the main text, we define the independent parameters (A, r, p) by the relation $(K, W, U, U') = (4A^2, 1, r, pr)$, for fitting the spin structure factor of the asymmetric field theory.

VIII. REAL-SPACE CORRELATIONS OF THE RANK-2 U(1) FIELD THEORY

Here, we calculate the real-space spin correlations $C(\boldsymbol{r})$ of the effective rank-2 U(1) field theory by analytically Fourier-transforming the spin structure factor derived in Eq. (32) of the last section,

$$C(\boldsymbol{r}) = \int \frac{d^2\boldsymbol{q}}{(2\pi)^2} \mathcal{S}(\boldsymbol{q}) e^{\imath \boldsymbol{q}\cdot\boldsymbol{r}}. \tag{34}$$

We are specifically interested in the type of spatial decay of the correlations at long distances, i.e., in the continuum limit at $\boldsymbol{q} \to 0$. For any finite W there is a range of small momenta where the first term $U/4$ in the second square root of the denominator in Eq. (32) dominates over the second term $\sim W$. In this regime $\mathcal{S}(\boldsymbol{q})$ becomes

$$\mathcal{S}(\boldsymbol{q}) = \sqrt{\frac{K}{U}} \frac{(L_1 - L_2)^2}{\sqrt{L_1^2 + L_2^2}}. \tag{35}$$

To make further progress by analytical calculations, we slightly modify the field theory of the last section and replace the first component of the constraint vector in Eq. (27) by

$$L_1(\boldsymbol{q}) = -4 s_x s_y \longrightarrow -2 s_x s_y. \tag{36}$$

This replacement is motivated by the fact that, in the limit $\boldsymbol{q} \to 0$, the norm of the constraint vector now depends solely on q and is independent of the polar angle,

$$L_1^2 + L_2^2 = q^4 \text{ at small } q, \tag{37}$$

making the analytical derivation of the polar angle integration in Eq. (34) possible. On the other hand, the radial dependence of $\mathcal{S}(\boldsymbol{q})$, and thus the decay behavior of real-space correlations, which is our primary interest here, remains unaffected. From a physical perspective, this replacement makes the photon dispersion rotation invariant at small momenta.

Inserting Eq. (35) into Eq. (34) and expanding the constraint vector in lowest order in q one obtains

$$\begin{aligned} C(\boldsymbol{r}) &\sim \int \frac{d^2\boldsymbol{q}}{(2\pi)^2} (q_x^2 - q_y^2 + 2 q_x q_y)^2 \frac{e^{\imath \boldsymbol{q}\cdot\boldsymbol{r}}}{q^2} \\ &= (\partial_x^2 - \partial_y^2 + 2\partial_x\partial_y)^2 \int \frac{d^2\boldsymbol{q}}{(2\pi)^2} \frac{e^{\imath \boldsymbol{q}\cdot\boldsymbol{r}}}{q^2} \\ &\equiv (\partial_x^2 - \partial_y^2 + 2\partial_x\partial_y)^2 g(\boldsymbol{r}). \end{aligned} \tag{38}$$

The function $g(\boldsymbol{r})$ defined in the last line obeys the relation

$$(\partial_x^2 + \partial_y^2) g(\boldsymbol{r}) = -\int \frac{d^2\boldsymbol{q}}{(2\pi)^2} e^{\imath \boldsymbol{q}\cdot\boldsymbol{r}} = -\delta(\boldsymbol{q}). \qquad (39)$$

This equation can be easily integrated, yielding $g(\boldsymbol{r}) \sim \log(|\boldsymbol{r}|)$, which results in the real-space correlations

$$C(\boldsymbol{r}) \sim \frac{xy(-x^2+y^2)}{|\boldsymbol{r}|^8}. \qquad (40)$$

Most importantly, $C(\boldsymbol{r})$ decays as $\sim \frac{1}{|\boldsymbol{r}|^4}$ along any radial direction.

IX. FURTHER INDICATIONS FOR A GAPLESS HIGHER-RANK SPIN LIQUID

The main text demonstrates that the phase diagram of the spin-1 spiderweb model displays a transition from a disordered, spin-liquid phase adiabatically connected to the solvable RK point to an ordered phase at finite μ_c, which can differ between sectors. This spin liquid phase was found to be well described by a rank-2 U(1) quantum gauge theory.

Here, we further investigate the nature of the pinch point suppression, discussed in the main text and derived in the previous section. Expressed in radial coordinates $q = |\boldsymbol{q}|$ and φ, close to the pinch point origin, i.e. at $\boldsymbol{q} = (0, 0)$, the spin structure factor from the field theory in Eq. (32) is of the form

$$\mathcal{S}(q, \varphi) \approx g(\varphi) q^2 \qquad (41)$$

where $g(\varphi)$ is a function of only the angle φ. It is thus insightful to rescale the spin structure factor by $1/q^2$, as this should reveal the singular nature of the fourfold pinch point. The result is shown for the 6×6 sector at $\mu = 0.8J'$ in Supplementary Fig. 5. For reference, panels (a-c) show a comparison of GFMC and field theory results for $\mathcal{S}(q, \varphi)$ without such rescaling for several circular paths around the pinch point. After rescaling $\mathcal{S}(\mathbf{q})/q^2$, in both GFMC, Supplementary Fig. 5(d) and field theory, Supplementary Fig. 5(e), the fourfold pinch point becomes visible. Moreover, the curves showing the cuts along the circular paths in Supplementary Fig. 5(f) collapse onto a single curve, confirming the expected form in Eq. (41). In order to better visualize this collapse without artifacts from the finite momentum space resolution, we also show a sinc-interpolated version of the GFMC data for $\mathcal{S}(q, \varphi)$ in panels (g,i). We find that this interpolation is the most natural, as it corresponds to a Fourier transform of an effectively enlarged system size, where long-distance correlations $\gtrsim L$ are neglected. Aside from artifacts at the smallest momenta (which correspond to inaccuracies of the vanishingly small long-range correlations), we find that this reproduces the continuum field theory shown in panel (h) exceptionally well.

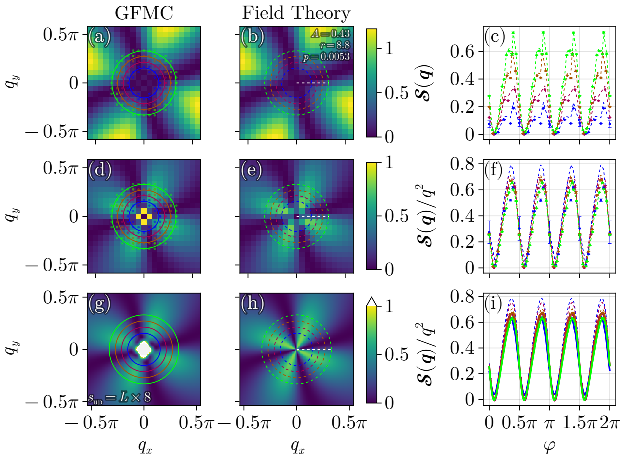

Supplementary Fig. 5. $\mathcal{S}(q, \varphi)$ close to the pinch point for $L = 36$ at $\mu = 0.8J'$ for GFMC (a,d,g) and field theory (b,e,h) in the 6×6 sector. Also shown are momentum discretized, circular paths with a starting point $\varphi = 0$ indicated by a white dashed line. (c,f,i): Circular cuts of $\mathcal{S}(q, \varphi)$ for the paths shown. (d,e,f) shows the rescaled quantity $\mathcal{S}(q, \varphi)/q^2$. (g): Same data in (d), but upsampled by a factor of 8 using sinc interpolation. The radius of white area at $\boldsymbol{q} = (0, 0)$ indicates the resolution limit $\sim 2\pi/L$ permitted by the finite system size, above which correlations are neglected. The field theory estimate (h) is not upsampled and has perfect resolution.

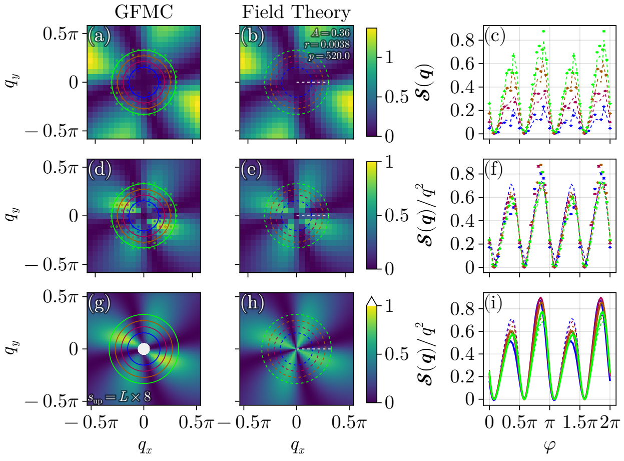

Supplementary Fig. 6. Same as in Supplementary Fig. 5 but in the diagonal stripe sector for $\mu = 0.9J'$.

We also repeat this analysis for the diagonal stripe sector at $\mu = 0.9J'$ and $\mu = 0.6J'$, respectively, in Figs. 6 and 7. In the spin-liquid phase at $\mu = 0.9J'$, the findings are largely equivalent, aside from a slightly enhanced asymmetry of the spin structure factor in the GFMC data. The observation of a spin structure factor of the form in Eq. (41) changes in the ordered phase at $\mu = 0.6J'$ of the diagonal stripe sector, where we find a much stronger suppression $\sim q^{5/2}$ in contrast to the best field-theory fit; see Supplementary Fig. 7. We note that

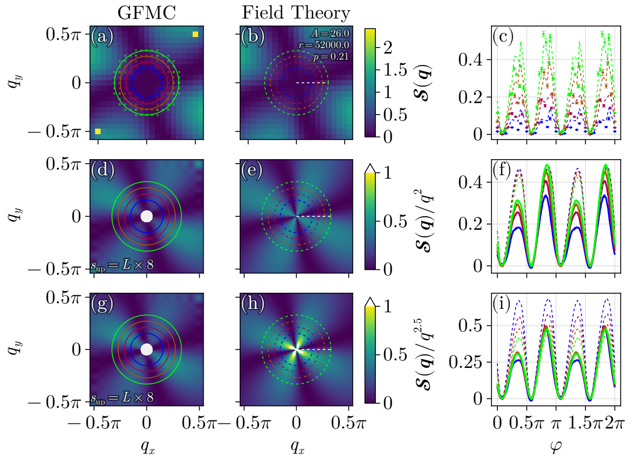

Supplementary Fig. 7. As in Supplementary Fig. 6, but in the ordered phase for $\mu = 0.6J'$. Panels (g-i) show the collapse for a structure factor rescaled by a higher power $1/q^{2.5}$.

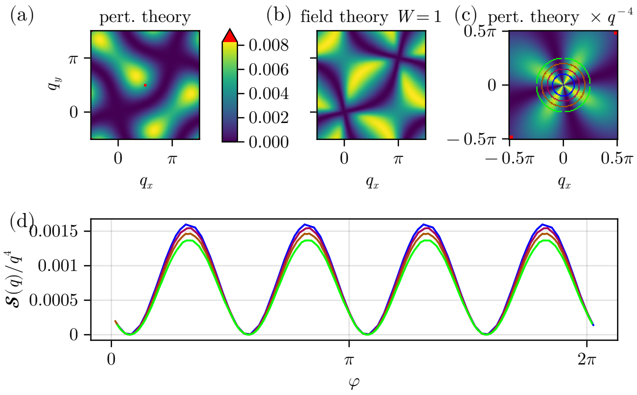

Supplementary Fig. 8. (a) Spin structure factor in the sector of the diagonal stripe state at $\mu \to -\infty$ computed in first order perturbation theory in $\mathcal{H}_2$. The color scale was truncated to not include the ordering peak so that the perturbative fluctuations are visible. (b) Prediction of the spin structure factor of the fractonic U(1) field theory of Eq. (32) at $W = 1$. (c) Same as (a) but rescaled by $\frac{1}{q^4}$ and plotted in a smaller part of the momentum space. (d) Spin structure factor in (c) plotted along the rings shown in (c).

the precise exponent of the scaling is difficult to obtain to high accuracy, rendering an estimation of the phase boundaries using this feature rather difficult.

A qualitative explanation of this stronger suppression is obtained by considering the perturbative limit of the stripe ordered state, denoted $|\psi_{\rm st}\rangle$, at $\mu \to -\infty$. Since $|\psi_{\rm st}\rangle$ is an eigenstate of $\mathcal{H}_1$ and $\mathcal{H}_3$ and since $\mathcal{H}_2$ is much smaller than $\mathcal{H}_1 + \mathcal{H}_3$ we treat the effect of $\mathcal{H}_2$ on $|\psi_{\rm st}\rangle$ perturbatively. In first order in $\mathcal{H}_2$, the perturbed state takes the form

$$|\psi^{(1)}\rangle = |\psi_{\rm st}\rangle - \frac{J'}{2\mu} \sum_{\square} \left(\mathcal{F}_{\square} + \mathcal{F}_{\square}^{\dagger}\right) |\psi_{\rm st}\rangle. \quad (42)$$

The spin structure factor $\mathcal{S}(\boldsymbol{q})$ of $|\psi^{(1)}\rangle$ shown in Supplementary Fig. 8(a) features magnetic Bragg peaks at $\boldsymbol{q} = \pm(\pi/2, \pi/2)$ indicative of diagonal stripe order. Furthermore, the spin structure factor exhibits a diffuse signal from the application of fluctuator moves $\mathcal{F}_{\square}$ and $\mathcal{F}_{\square}^{\dagger}$ on $|\psi_{\rm st}\rangle$. This diffuse signal has the analytical form

$$\mathcal{S}_{\rm diff}(\boldsymbol{q}) \sim [\cos(q_x) - \cos(q_y) + 2\sin(q_x)\sin(q_y)]^2. \quad (43)$$

Interestingly, $\mathcal{S}_{\rm diff}(\boldsymbol{q})$ also has a characteristic structure of nodal features and suppressed pinch points. However, its precise shape is different from the spin structure factor of our fractonic U(1) gauge theory shown for $W = 1$ in Supplementary Fig. 8(b) for comparison (see Eq. (32) for the analytical expression). Specifically, as shown in Supplementary Fig. 8(c) and (d), this first-order spin structure factor scales as q^4 for small momenta, explaining the observed suppression stronger than q^2 once magnetic long-range order sets in.

[1] N. Niggemann, M. Adhikary, Y. Schaden-Thillmann, and J. Reuther, Classical fracton spin liquid and Hilbert space fragmentation in a 2D spin-1/2 model, Phys. Rev. B **113**, 165118 (2026).
[2] P. Sala, T. Rakovszky, R. Verresen, M. Knap, and F. Pollmann, Ergodicity Breaking Arising from Hilbert Space Fragmentation in Dipole-Conserving Hamiltonians, Phys. Rev. X **10**, 011047 (2020).
[3] A. Khudorozhkov, A. Tiwari, C. Chamon, and T. Neupert, Hilbert Space Fragmentation in a 2D Quantum Spin System with Subsystem Symmetries, SciPost Physics **13**, 098 (2022).
[4] C. Stahl, O. Hart, A. Khudorozhkov, and R. Nandkishore, Strong Hilbert Space Fragmentation and Fractons from Subsystem and Higher-Form Symmetries (2025), arXiv:2505.15889 [cond-mat].
[5] D. Adler, D. Wei, M. Will, K. Srakaew, S. Agrawal, P. Weckesser, R. Moessner, F. Pollmann, I. Bloch, and J. Zeiher, Observation of Hilbert space fragmentation and fractonic excitations in 2D, Nature **636**, 80 (2024).
[6] M. Will, R. Moessner, and F. Pollmann, Realization of Hilbert Space Fragmentation and Fracton Dynamics in

Two Dimensions, Phys. Rev. Lett. **133**, 196301 (2024).
[7] X. Feng and B. Skinner, Hilbert space fragmentation produces an effective attraction between fractons, Phys. Rev. Res. **4**, 013053 (2022).
[8] K. Lee, A. Pal, and H. J. Changlani, Frustration-induced emergent Hilbert space fragmentation, Physical Review B **103**, 235133 (2021).
[9] M. Hermele, M. P. A. Fisher, and L. Balents, Pyrochlore photons: The $U(1)$ spin liquid in a $S = \frac{1}{2}$ three-dimensional frustrated magnet, Phys. Rev. B **69**, 064404 (2004).
[10] L. Savary and L. Balents, Quantum spin liquids: a review, Rep. Prog. Phys. **80**, 016502 (2016).
[11] M. Pretko, Generalized electromagnetism of subdimensional particles: A spin liquid story, Phys. Rev. B **96**, 035119 (2017).
[12] J. Colpa, Diagonalization of the quadratic boson hamiltonian, Physica A: Statistical Mechanics and its Applications **93**, 327 (1978).
