# Supplementary material for: Gapless fracton quantum spin liquid and emergent photons in a 2D spin-1 model
Source: Nat Commun. 2026 Jul 11;17:6131. doi: 10.1038/s41467-026-74797-0 (PMC13365202; doi:10.1038/s41467-026-74797-0)
Supplement: Supplementary file 2 — Transparent Peer Review file [file 41467_2026_74797_MOESM2_ESM.pdf]

nature portfolio

Peer Review File

Gapless fracton quantum spin liquid and emergent photons in a 2D spin-1 model

Corresponding Author: Dr Nils Niggemann

This file contains all reviewer reports in order by version, followed by all author rebuttals in order by version.

Version 0:

Reviewer comments:

Reviewer #1

(Remarks to the Author)
The paper “gapless fraction quantum spin liquid and emergent photons in a 2D spin-1 model” by Nils Niggemann et al. is a very interesting and timely study. The authors analyze a spin-1 model with local constraints and use GFMC to map out ground-state and excited-state properties. They identify a finite window with no magnetic long-range order, and a structure factor showing fourfold pinch points that match a rank-2 U(1) gauge-theory prediction with quantitatively decent agreement. The work offers an interesting and controlled microscopic spin model for a gapless fracton-type spin liquid.

I would be happy to recommend publication in Nature Communication provided the authors address a few clarifications:

(1) In the companion work of the authors [arxiv:2508.06606], the spin-1/2 version of the same model has severe Hilbert-space fragmentation, yielding either long-range order or a classical fracton liquid even near the RK point. Here, by contrast, the authors claim the spin-1 case a quantum spin liquid, where quantum fluctuation removes the long-range order, supported by absence of peaks in S(q), and fits to a rank-2 U(1) field theory. As I understand it, the absence of QSL in the spin-1/2 version is that the Hilbert-space fragmentation is hindering a coherent superposition of configurations which removes the magnetic order; while in the spin-1 version there are much more states connected to the diagonal stripe state in Fig. 3(a6), whereby the quantum fluctuations is enough to remove the order. Here is it possible to quantify the “milder fragmentation” (distributions of sector sizes/move-graph connectivity), which suffices to suppress the magnetic order in comparison to spin-1/2 case.

(2) The putative effective rank-2 field theory there is a clear pinch point structure which matches the numerical results. However, it seems that both the spin-1 model here and the previous spin-1/2 companion paper [arxiv:2508.06606] show pinch-point patterns consistent with the effective rank-2 U(1) field theory. This is always true on a classical level of Gaussian approximation. Since those pinch points also appear qualitatively in the spin-1/2 case, what precisely distinguishes the spin-1 phase you call a QSL from the spin-1/2 phase you classify as classical / not-QSL? Is it just by the additional evidence on the absence of long-range order in the range of 0.81 < mu/J’ < 1? Or if other evidence regarding thermal dynamics / fracationalization / emergent gauge field also relevant? -- this is also related to the next question.

(3) A question regarding the scope of the term “QSL”. In the more conventional (non-fracton) usage in the QSL community, QSL typically implies fractionalization (e.g., the Kitaev Z2 or U(1) Dirac QSL with fractionalized spinons supported by VQMC). In this work, the designation appears to rest on the absence of magnetic order and a close fit to a putative rank-2 U(1) field theory, without direct mentioning fractionalized matter or long-range entanglement. Could the authors clarify in what precise sense they are using “QSL” here, i.e., is the claim a phenomenological quantum Coulomb–like phase (photon), or a stronger claim involving fractionalization/long-range entanglement? If the latter, it would be good to discuss what support it or why such diagnostics are presently out of reach.

A few Minor things:

On the Gaussian constraints: the Gaussian approach treats spins as unconstrained spins subjected to C_\square = 0. This is one of the essential statement for the rank-2 Gauss law in the approximation. This is beautiful. But it is not that immediately clear and I admit it took me a while to appreciate this. Later I realized it is stated in the other work of the same group [Ref. 47]; A short note in the current manuscript on how this is derived in method section or supplemental would be helpful.

In analyzing the classical fracton configuration in Sec III B, the coexistence of immobile, partially mobile and fully mobile particles is common in gauge models with subsystem symmetries. As is also mentioned by the author in subsection III C on subdimensional constants of motion. Here it may be relevant to cite the original work that reported such dimensional reduction physics in classical gauge-like models [Xu and Moore, Phys. Rev. Lett. 93, 047003 (2004)].

(Remarks on code availability)

Reviewer #2

(Remarks to the Author)
Dear Editor,

The authors introduce and study a spin-1 model on a square lattice (spiderweb model). Using the Green's function Monte Carlo method, they try to identify a gapless fracton quantum spin liquid, mainly concerning the static spin structure factor S(q). A central novelty of the paper is the observation of emergent photons, supported by the agreement between S(q) and a rank-2 U(1) field theory prediction.

The paper is well organized and well written, the results are interesting, and the topic is exotic. Nevertheless, I have several issues list below:
1. The authors claim that the low-energy physics is described by a field theory with a photon dispersion ω(q)~q^2, implying that S(q) ~ q^2 as q → 0. While Fig. 5(c) clearly shows S(q) vanishing as q →0, I am concerned that this observation alone does not fully confirm the emergence of photons. For a pinch point singularity, S(q) is highly anisotropic and vanishes along some possible nodal directions even in the trivial classical limit. Since the quantum scenario requires S(q) ~ q^2 along all directions in the BZ, it will be much better if the authors can show more path, such as Γ→ (π, π) and/or other directions.
2. I note the drastic change of S(q) between μ=0.8J′ and μ=0.81J′. This suggests a first-order phase transition between magnetic ordered and QSL? Does the proposed field theory predict a first-order transition? A phase transitions from the U(1) QSL to AFM, often described by Higgs mechanisms, is usually continuous The authors should clarify this discrepancy.
3. The authors argue that the suppression of S(q) is the strong evidence for the emergent photon. Nevertheless, these same nodal features persist in the ordered phase, the dark nodal regime around (0,0) and (π, π) when μ=0.8J′, see Fig. 4(c). It implies that they are likely consequences of the local kinematic constraints (maybe, a generalized ice rules..) satisfied by the spins, instead of quantum dynamical photon mode. The authors need to address why this feature should be considered a strong evidence for the QSL when it is also present in the trivial phase.
4. In Section V, the authors briefly mention the stability of the gapless phase against U(1) gauge fluctuations. It is well-established that in compact U(1) gauge theories in 2+1 dimensions, the monopoles can generate a mass gap for the photon, e.g., a confined phase. I would suggest the authors to carry out a careful scaling analysis in their Monte Carlo simulations to justify the gapless ground state.
5. Even though it is a pure theoretical paper, it will be much better to discuss how to realize this interesting model in realistic systems? Such as Rydberg atoms?

Once the authors address all the issues, I would recommend publication

(Remarks on code availability)

Version 1:

Reviewer comments:

Reviewer #1

(Remarks to the Author)
I thank the authors for their detailed and constructive response. In my view, they have adequately addressed my main concerns, in particular by clarifying the distinction between the spin-1 and spin-1/2 cases, sharpening the meaning of quantum spin liquid in their context. Although the microscopic model remains somewhat engineered and contrived, I find the physical mechanism interesting and the results potentially valuable for the broader study of fractonic gauge structures and quantum spin liquids. I therefore recommend publication.

(Remarks on code availability)

Reviewer #2

(Remarks to the Author)
I have carefully read the revised manuscript. I was satisfied with most of the authors' job addressing the reviewers' concerns.

I have only one minor observation regarding the discussion of the $q^{2.5}$ versus q^2 scaling added in the SM. While the sinc interpolation creates visually pleasing continuous circular cuts, I think that Distinguishing $q^{2.5}$ from q^2 based purely on finite-size numerical data requires extreme caution.

However, I understand and accept the authors' underlying physical argument: the suppression of the nodal features in the ordered phase is somehow faster than q^2. Therefore, the physical picture sounds and I have no further questions.

I would like to recommend the manuscript for publication.

(Remarks on code availability)

Open Access This Peer Review File is licensed under a Creative Commons Attribution 4.0 International License, which permits use, sharing, adaptation, distribution and reproduction in any medium or format, as long as you give appropriate credit to the original author(s) and the source, provide a link to the Creative Commons license, and indicate if changes were made.
In cases where reviewers are anonymous, credit should be given to 'Anonymous Referee' and the source.
The images or other third party material in this Peer Review File are included in the article's Creative Commons license, unless indicated otherwise in a credit line to the material. If material is not included in the article's Creative Commons license and your intended use is not permitted by statutory regulation or exceeds the permitted use, you will need to obtain permission directly from the copyright holder.
To view a copy of this license, visit https://creativecommons.org/licenses/by/4.0/

Response to Referee Reports

Manuscript: *Gapless fracton quantum spin liquid and emergent photons in a 2D spin-1 model*
Authors: *Nils Niggemann, Meghadeepa Adhikary, Yannik Schaden-Thillmann, Johannes Reuther*
Date: April 14, 2026

Dear Editor,

We thank the referees for their positive assessment of our manuscript and their endorsement for publication in *Nature Communications*. We are grateful for their constructive feedback, which we have addressed to improve our manuscript. Changes to the main text are indicated in blue. In addition, we have included new Sections I, IV, VIII, and IX in the Supplementary Material. Below, we address their individual questions and suggestions, with each referee comment quoted and our response following.

Response to Reviewer #1

We thank Reviewer #1 for their positive evaluation and for recommending publication. We address their clarifications below.

Referee Comment

(1) In the companion work of the authors [arxiv:2508.06606], the spin-1/2 version of the same model has severe Hilbert space fragmentation, yielding either long-range order or a classical fracton liquid even near the RK point. Here, by contrast, the authors claim the spin-1 case a quantum spin liquid, where quantum fluctuation removes the long-range order, supported by absence of peaks in S(q), and fits to a rank-2 U(1) field theory. As I understand it, the absence of QSL in the spin-1/2 version is that the Hilbert space fragmentation is hindering a coherent superposition of configurations which removes the magnetic order; while in the spin-1 version there are much more states connected to the diagonal stripe state in Fig. 3(a6), whereby the quantum fluctuations is enough to remove the order. Here is it possible to quantify the ”milder fragmentation” (distributions of sector sizes/move-graph connectivity), which suffices to suppress the magnetic order in comparison to spin-1/2 case.

Response:

The Referee raises an interesting question regarding the quantification of the fragmentation in the spin-1/2 and spin-1 cases. To address this point, we have added further analysis to our manuscript in the Supplementary Material, which we also summarize here.

Utilizing the large number of local constraints, it is possible to explicitly construct a Hamiltonian as a matrix within the full fracton-free Hilbert space, i.e., up to $L = 8$ for spin-1/2 and $L = 6$ for spin-1[1]. Treating the Hamiltonian as an adjacency matrix, we analyze the connectivity structure

[1] While it is possible to count the number of fracton-free configurations for even larger system sizes, analyzing the size of Hilbert space sectors is severely limited by memory requirements. For example, just storing the approximately 4.7×10^{10} solutions for $L = 8, S = 1$ would require around ~ 300 GB of memory, accounting for time-reversal and substantial compression of configurations.

of the resulting move graph to quantify the fragmentation of the Hilbert space. The results are summarized in Table 1.

We define the fragmentation quotient

$$f = (n_{\text{sectors}}/n_{\text{total}})^{1/L^2}, \tag{1}$$

where n_{total} is the total number of fracton-free spin states and n_{sectors} is the number of Hilbert space sectors for a given system size L. For a fully fragmented Hilbert space, the number of sectors equals the size of the Hilbert space, i.e., $n_{\text{sectors}} = n_{\text{total}}$, and thus $f = 1$. On the other hand, if there is no fragmentation, the number of sectors n_{sectors} grows less than exponentially with the system size L^2, implying $f \to 0$ as $L \to \infty$. For spin-1/2, we find $f \approx 0.98$, which is close to the fully disconnected case $f = 1$. The corresponding value for spin-1 is $f \approx 0.93$, indicating a much milder but still significant fragmentation. The reduced fragmentation has a more drastic effect on the average connectivity relative to the system size, which increases by a factor of ~ 5 from spin-1/2 to spin-1 for $L = 6$.

L	Total number of states		Sectors		f		avg. connectivity / L^2	
	Spin-1/2	Spin-1	Spin-1/2	Spin-1	Spin-1/2	Spin-1	Spin-1/2	Spin-1
4	216	6861	184	2621	0.99	0.942	0.01875	0.1025
6	5912	5800829	3732	473717	0.987	0.933	0.0225	0.1222
8	350872	47067992003	108534	—	0.982	—	0.0339	—
10	37403668	—	6042418	—	0.982	—	0.0319	—
12	10103561614	—	—	—	—	—	—	—

Table 1: Sector analysis for spin-1/2 and spin-1 on $L \times L$ lattices. $f = (\text{Sectors}/\text{Total})^{1/L^2}$ indicates the relative system-size scaling of sectors with respect to total solutions. Also listed is the average connectivity per L^2. Dashes indicate data that was infeasible to obtain due to memory requirements.

This interpretation is further supported by Fig. 1(a), showing the total sizes of Hilbert space sectors for $L = 6$. Compared to spin-1/2 (red), the Hilbert space of spin-1 (black) has a much broader distribution with significant numbers of large sectors.

The effects are also quantifiable within a single sector: Fig. 1(b) shows the distribution of node connectivities in the spin-1/2 staircase (red) and spin-1 diagonal stripe (black) sectors stochastically sampled for a larger system size $L = 28$. Both represent highly connected sectors compared to the average listed in Tab. 1. For spin-1/2, connectivities are much lower and more narrowly distributed around the mean compared to spin-1.

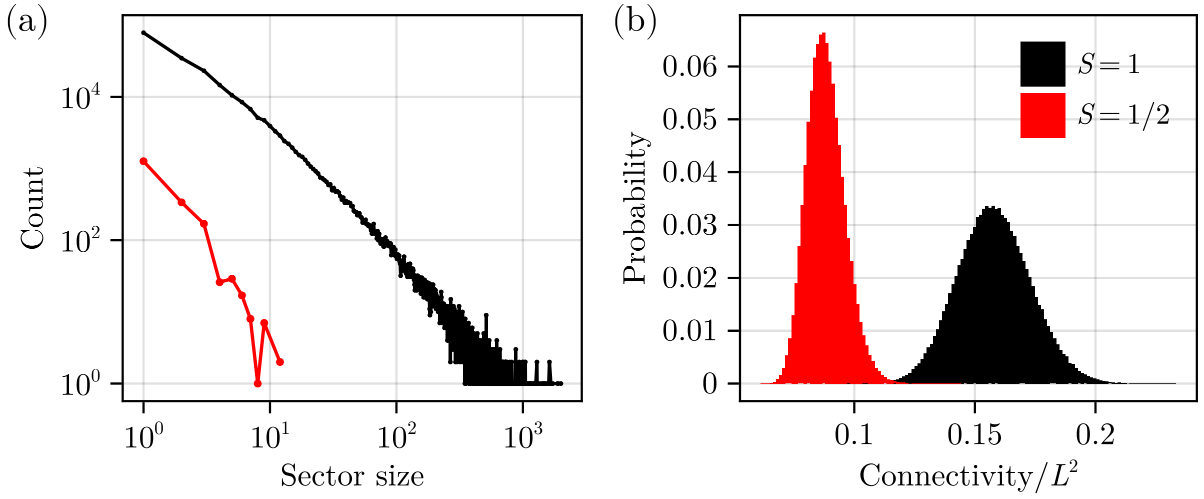

Figure 1: (a) Histogram of connected-sector sizes for spin-1/2 and spin-1 for $L = 6$ obtained via exact enumeration of solutions. (b) Probability distribution of connectivity of configurations in the staircase (spin-1/2) and diagonal stripe (spin-1) sector for $L = 28$, obtained via GFMC sampling of configurations at the RK point.

Referee Comment

(2) The putative effective rank-2 field theory there is a clear pinch point structure which matches the numerical results. However, it seems that both the spin-1 model here and the previous spin-1/2 companion paper [arxiv:2508.06606] show pinch-point patterns consistent with the effective rank-2 U(1) field theory. This is always true on a classical level of Gaussian approximation. Since those pinch points also appear qualitatively in the spin-1/2 case, what precisely distinguishes the spin-1 phase you call a QSL from the spin-1/2 phase you classify as classical / not-QSL? Is it just by the additional evidence on the absence of long-range order in the range of $0.81 < \mu/J' < 1$? Or if other evidence regarding thermal dynamics / fracationalization / emergent gauge field also relevant? – this is also related to the next question.

Response:

We thank the referee for this important question that clarifies the distinction between classical and quantum fracton spin liquids.

The key difference is the realization of a *gapless* state with emergent photons *in a single ergodic sector*. Indeed, working with a less strict condition, which does not take the fragmented Hilbert space into account, one might be tempted to also refer to the spin-1/2 model as a quantum fracton spin liquid. Importantly, the spin-1/2 system has only pinch points if one averages over all possible sectors. Individually, each sector fails to recover the power-law correlations seen in a gapless state. This becomes most visible at the RK point, where a quantum fracton spin liquid should display intact, unsuppressed pinch points. By contrast, the sector containing the staircase state shown in the spin-1/2 companion paper shows magnetic ordering incompatible with power-law decay even

at the RK point. The true quantum ground state of this model, at any $\mu \neq J'$, is a superposition within a single sector with no fluctuations outside this sector. Even at the RK point, the ground state features no coherent quantum fluctuations between sectors, such that the state is better referred to as a classical spin liquid.

In the spin-1 case, fragmentation still exists; however, a gapless state with excellent agreement with the quantum rank-2 U(1) field theory is realized already at the level of individual sectors (see also our response to the fourth question of the second Referee and Fig. 7). Further evidence for the spin-liquid nature at finite $\mu \leq J'$ is provided in response to the other questions below.

We have added a brief comment in Sec. IV.A in our manuscript that addresses this question.

Referee Comment

(3) A question regarding the scope of the term "QSL". In the more conventional (non-fracton) usage in the QSL community, QSL typically implies fractionalization (e.g., the Kitaev Z2 or U(1) Dirac QSL with fractionalized spinons supported by VQMC). In this work, the designation appears to rest on the absence of magnetic order and a close fit to a putative rank-2 U(1) field theory, without direct mentioning fractionalized matter or long-range entanglement. Could the authors clarify in what precise sense they are using "QSL" here, i.e., is the claim a phenomenological quantum Coulomb–like phase (photon), or a stronger claim involving fractionalization/long-range entanglement? If the latter, it would be good to discuss what support it or why such diagnostics are presently out of reach.

Response:

We thank the Referee for this question. In fact, we adopt the standard definition of a quantum spin liquid used in the community, based on the existence of fractionalized excitations. While fractionalization was not strongly emphasized in the original manuscript, it is indeed present in our system. As illustrated in Fig. 1(d) of the manuscript, a single spin flip generates four fractons, i.e., four eight-site clusters in which the local constraint is violated. In this sense, a fracton can be viewed as carrying one quarter of a spin-flip excitation. This is analogous to spin ice, where a single spin flip creates two defect tetrahedra. Thus, fractionalization is already explicitly encoded in the classical part $\mathcal{H}_1$ of the spiderweb model, and we have now highlighted this point more clearly in the revised manuscript.

In the context of our work, one may apply an even more restrictive definition of a quantum spin liquid, where it is identified as a realization of a deconfined phase of a gauge theory in a spin system. This perspective encompasses all commonly discussed quantum spin liquids, including those mentioned by the Referee (e.g., Kitaev $\mathbb{Z}_2$ and U(1) Dirac spin liquids). These phases differ in the nature of their gauge fields, matter fields, and gauge constraints. Importantly, this sharper definition inherently implies fractionalization, since a gauge theory necessarily contains both gauge and matter degrees of freedom. Consequently, in a quantum spin liquid the microscopic spin degrees of freedom must fractionalize into these emergent fields. In comparison to more conventional $\mathbb{Z}_2$ or U(1) quantum spin liquids, our system just realizes a special and somewhat unusual type of gauge theory in which the matter fields (spinons) are fractons, characterized by their restricted mobility. Our claim that this gauge structure emerges in the spiderweb model is strongly supported by the excellent agreement between numerically computed spin structure factors and the predictions of

the field theory (see Figs. 5 and 6 in the manuscript).

The Referee also raises the question of long-range entanglement, another property often associated with quantum spin liquids. However, long-range entanglement is primarily a defining feature of *gapped* quantum spin liquids with topological order, such as the toric code. In such systems, it appears as a constant correction to the area law for entanglement entropy (the so-called topological entanglement entropy). By contrast, our system is *gapless*, due to the presence of gapless photon excitations (see also our response to Referee 2, Question 4). In gapless quantum spin liquids, the structure of entanglement is more intricate and harder to classify. For instance, in conventional quantum spin ice with gapless photons, the long-range entanglement is a logarithmic correction in system size, $\sim \log L$, see PRB 94, 125112 (2016). In a similar way, we also expect characteristic properties of long-range entanglement in our system. However, within the Green's function Monte Carlo approach employed here, the entanglement entropy is not directly accessible. For this reason, we do not attempt to characterize our system via entanglement measures, and instead focus on more direct signatures such as fractionalization and quantitative agreement with the corresponding gauge theory.

Minor comment 1

On the Gaussian constraints: the Gaussian approach treats spins as unconstrained spins subjected to C_square = 0. This is one of the essential statement for the rank-2 Gauss law in the approximation. This is beautiful. But it is not that immediately clear and I admit it took me a while to appreciate this. Later I realized it is stated in the other work of the same group [Ref. 47]; A short note in the current manuscript on how this is derived in method section or supplemental would be helpful.

Response:

We thank the referee for this suggestion and have added a discussion of this derivation to our Supplementary Material.

Minor comment 2

In analyzing the classical fracton configuration in Sec III B, the coexistence of immobile, partially mobile and fully mobile particles is common in gauge models with subsystem symmetries. As is also mentioned by the author in subsection III C on subdimensional constants of motion. Here it may be relevant to cite the original work that reported such dimensional reduction physics in classical gauge-like models [Xu and Moore, Phys. Rev. Lett. 93, 047003 (2004)].

Response:

We thank the Referee for pointing out this work, which we now reference in our revised manuscript.

Response to Reviewer #2

We thank Reviewer #2 for their positive assessment of our work and their detailed questions. We address each point below.

Referee Comment

1. The authors claim that the low-energy physics is described by a field theory with a photon dispersion $\omega(q) \sim q^2$, implying that $S(q) \sim q^2$ as $q \to 0$. While Fig. 5(c) clearly shows $S(q)$ vanishing as $q \to 0$, I am concerned that this observation alone does not fully confirm the emergence of photons. For a pinch point singularity, S(q) is highly anisotropic and vanishes along some possible nodal directions even in the trivial classical limit. Since the quantum scenario requires $S(q) \sim q^2$ along all directions in the BZ, it will be much better if the authors can show more path, such as $\Gamma \to (\pi, \pi)$ and/or other directions.

Response:
We agree with the Referee that it is insightful to validate the $\sim q^2$ scaling of the structure factor along other directions.

To address this in our manuscript, we extend our analysis to probe *each possible direction* by taking circular cuts of $S(\boldsymbol{q})$ for a variety of radii around the pinch point, also shown here in Fig. 2. Panels (a-c) show the comparison between GFMC and field theory in the diagonal stripe sector. Besides slight "step-like" features in the GFMC data, which are due to the discrete nature of the momentum space on finite systems, we again find good agreement between GFMC and field theory. The cuts displayed in Fig. 2(c) show increasing amplitudes of $S(\boldsymbol{q})$ for increasing $q = |\boldsymbol{q}|$. We further display $\mathcal{S}(q, \varphi)/q^2$, where q, φ are the radial and angular coordinates, in Fig. 2(d-f). In accordance with field-theoretical expectations, this scaling restores the singular nature of the fourfold pinch point, visible in panels (d) and (e). While the diagonal stripe sector features a slightly enhanced asymmetry of the pinch points compared to the field-theory fit, the collapse of the data for different q in panel (f) is compatible with the expected scaling relation $\mathcal{S}(q, \varphi) \sim q^2 g(\varphi)$ for a function $g(\varphi)$ that depends only on the angle. We note that a perfect collapse is only expected in the limit $q \to 0$, which requires very precise measurements of vanishingly small, long-range correlations.

In order to better visualize the scaling behavior, we also show a sinc interpolation of the GFMC data for $\mathcal{S}(q, \varphi)/q^2$ in Fig. 2(g). Formally, this corresponds to artificially enlarging the system by a factor of $s_{\mathrm{up}} = 8$ in each direction and re-computing the Fourier transform on this enlarged system, setting spin correlations for distances longer than the original system size to zero. Naturally, this approach likewise cannot truly increase the resolution around the pinch point (indicated by a white circle around the pinch point) which is characterized by slowly decaying correlations, but it allows us to obtain a smoother curve for larger q. Again, we see a clear collapse of the curves for different radii in Fig. 2(i). Deviations from a perfect collapse mainly occur at larger q which are expected since the small q limit no longer holds. Finally, we also perform the same analysis in the 6×6 sector shown in Fig. 3, where, at momenta away from the resolution limit $\sim 2\pi/L$, an excellent scaling collapse is found.

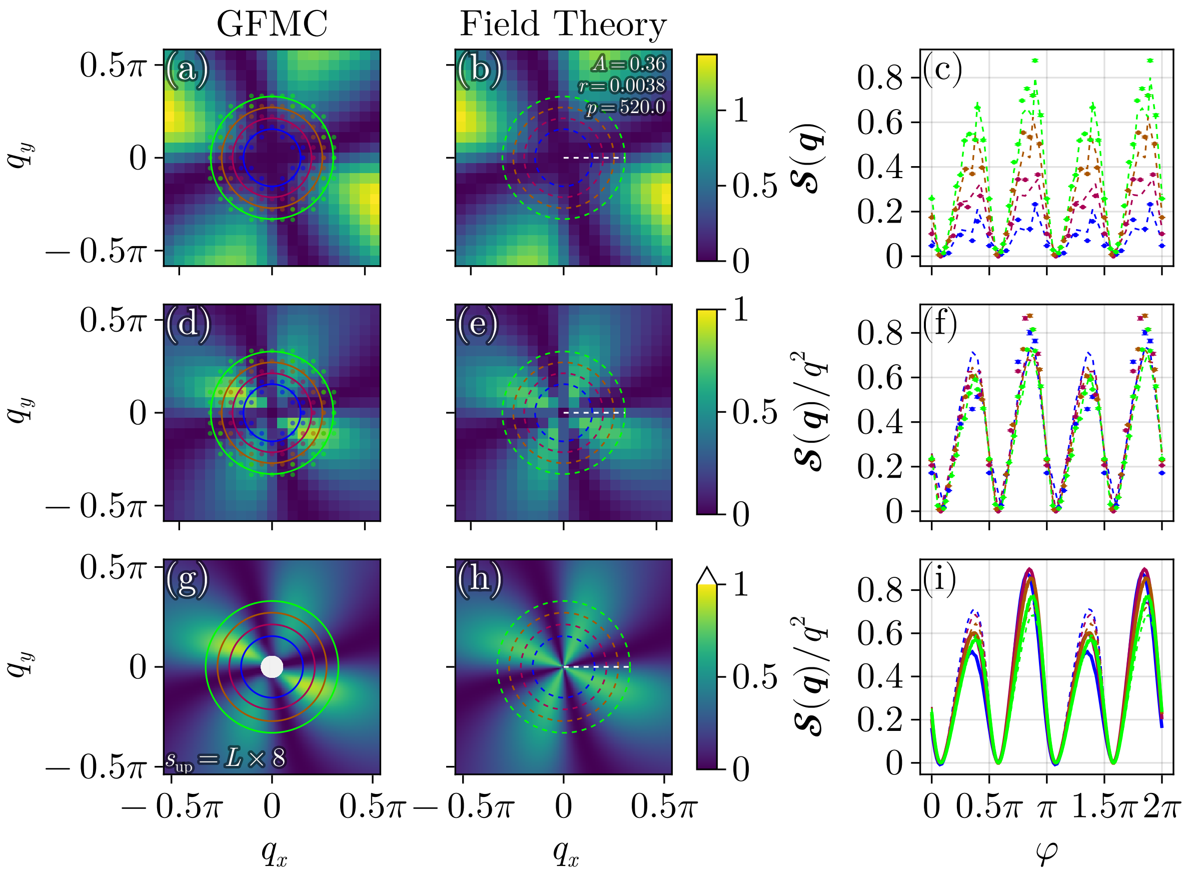

Figure 2: $\mathcal{S}(q,\varphi)$ close to the pinch point for $L = 36$ at $\mu = 0.9J'$ for GFMC (a,d,g) and field theory (b,e,h) in the diagonal stripe sector. Also shown are momentum-discretized circular paths (starting point $\varphi = 0$ indicated by a white dashed line). (c,f,i): Circular cuts of $\mathcal{S}(q,\varphi)$ for the paths shown. (d,e,f) shows the rescaled quantity $\mathcal{S}(q,\varphi)/q^2$. (g): Same data in (d), but upsampled by a factor of 8 using sinc interpolation. The radius of the white circle at $\boldsymbol{q} = (0,0)$ indicates the resolution limit $\sim 2\pi/L$ permitted by the finite system size, above which correlations are neglected. The field theory estimate (h) is not upsampled and has perfect resolution.

Referee Comment

2. I note the drastic change of S(q) between $\mu = 0.8J'$ and $\mu = 0.81J'$. This suggests a first-order phase transition between magnetic ordered and QSL? Does the proposed field theory predict a first-order transition? A phase transitions from the U(1) QSL to AFM, often described by Higgs mechanisms, is usually continuous The authors should clarify this discrepancy.

Response:

Thank you for raising this important question regarding the order of the phase transition between the QSL phase and the magnetically ordered phase. Indeed, as shown in Fig. 4(b) in the manuscript, the order parameter (related to the peak height in the spin structure factor) exhibits a pronounced

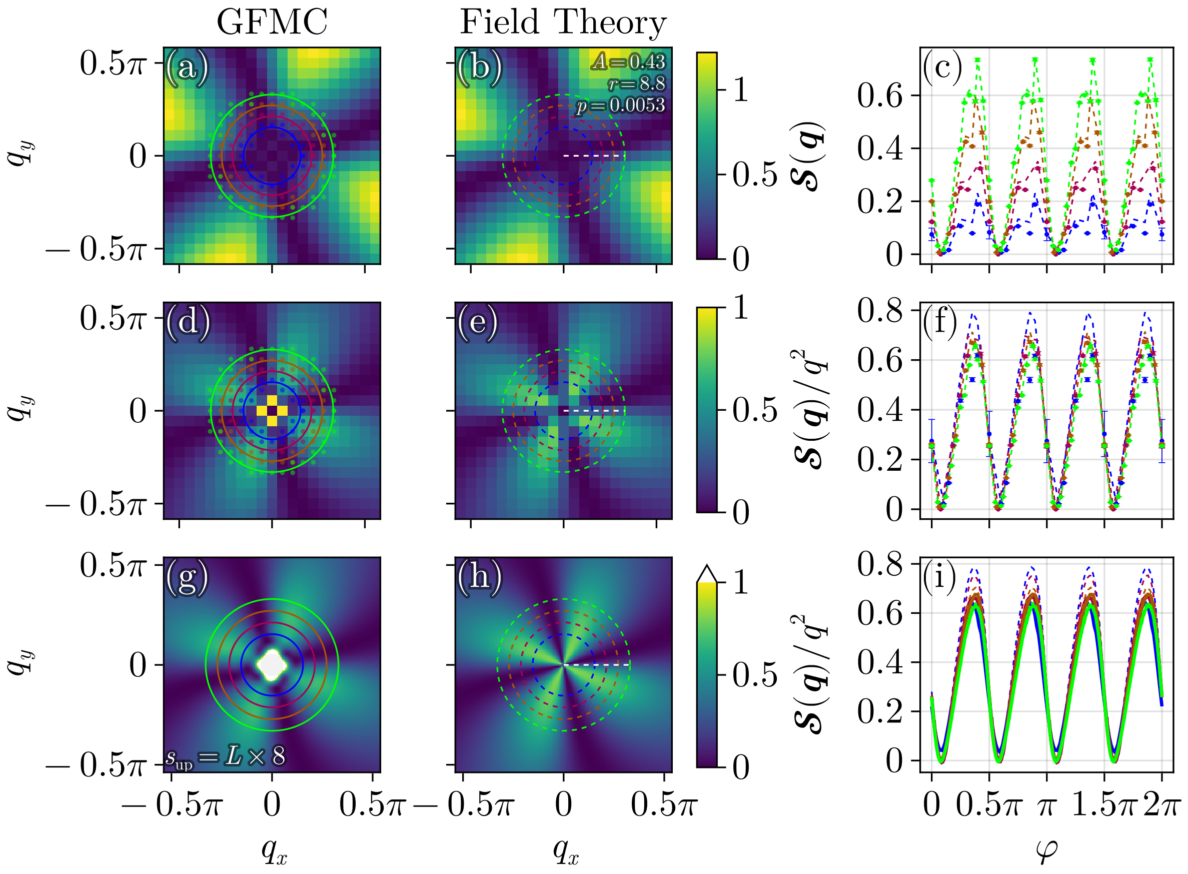

Figure 3: Same as in Fig. 2 but in the 6×6 sector for $\mu = 0.8J'$.

jump that becomes more pronounced with increasing system size, which is indicative of a first-order transition. However, we do not believe this observation contradicts general theoretical expectations.

To clarify the situation, it is crucial to distinguish between the Higgs phase (driven by a Higgs mechanism, and likely what the Referee had in mind) and the confined phase. A compact U(1) gauge theory is expected to exhibit a qualitative phase diagram of the type sketched in Fig. 4, applicable both to conventional rank-1 QED and to our rank-2 generalization. The horizontal axis represents a coupling that reduces the monopole gap while leaving the charge (fracton) gap finite. A concrete example for this coupling is the negative Rokhsar–Kivelson potential $-\mu$, which energetically favors ordered configurations with many flippable plaquettes but does not close the charge gap. The vertical axis corresponds to a coupling that reduces the charge gap, such as a transverse interaction $J_\perp(S_i^x S_j^x + S_i^y S_j^y)$ or a transverse field $h_\perp S_i^x$, which can drive charge condensation and realize a Higgs mechanism. The resulting phase diagram contains three phases: the deconfined (Coulomb) phase when both couplings are small, a confined phase upon increasing $-\mu$, and a Higgs phase upon increasing $J_\perp$ or $h_\perp$. (We note in passing that in conventional compact QED in 3+1 dimensions, the confined and Higgs phases are continuously connected via the Fradkin–Shenker continuity; see Phys. Rev. D 19, 3682 (1979).)

The Referee appears to associate the transition shown in Fig. 4 in the manuscript with a Higgs

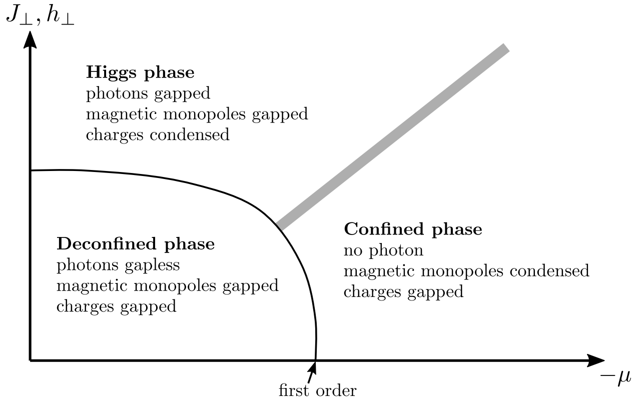

Figure 4: Schematic phase diagram of compact U(1) gauge theories.

transition along the vertical axis. However, the transition we observe is instead a confinement transition along the horizontal axis, since the fractons remain gapped throughout this parameter regime (we simulate a pure gauge theory in fracton-free sectors, effectively assuming an infinite fracton mass gap). The confinement transition along the horizontal axis has been extensively studied in conventional compact QED in 3+1 dimensions, where it has been established to be weakly first order (see e.g. Phys. Lett. B 133, 103-107 (1983), Phys. Lett. B 424, 328-334 (1998), Phys. Lett. B 604, 82-90 (2004)). Therefore, our observation of a first-order confinement transition in a compact rank-2 QED is not contradictory to the expectations from conventional U(1) gauge theories and is, in fact, fully consistent with them. We agree with the Referee, however, that the situation can differ at the Higgs transition, which (depending on the specific coupling driving it) may be either first or second order.

Finally, the Referee asks what our proposed field theory predicts regarding the order of the transition. Our field theory is a free Maxwell-type theory that retains only the leading-order term in an expansion of the gauge potential. Phase transitions out of this free theory necessarily involve non-perturbative physics beyond such an expansion, and their properties therefore cannot be inferred from the field theory alone. Addressing these transitions requires non-perturbative methods, which is precisely the role played by our numerical simulations.

We have added a brief comment to the manuscript in Sec. IV.B clarifying that the first-order confinement transition we observe is directly analogous to the first-order confinement transition known from conventional compact QED in 3+1 dimensions.

Referee Comment

3. The authors argue that the suppression of S(q) is the strong evidence for the emergent photon. Nevertheless, these same nodal features persist in the ordered phase, the dark nodal regime around $(0, 0)$ and (π, π) when $\mu = 0.8J'$, see Fig. 4(c). It implies that they are likely consequences of the local kinematic constraints (maybe, a generalized ice rules..) satisfied by the spins, instead of quantum dynamical photon mode. The authors need to address why this feature should be considered a strong evidence for the QSL when it is also present in the trivial phase.

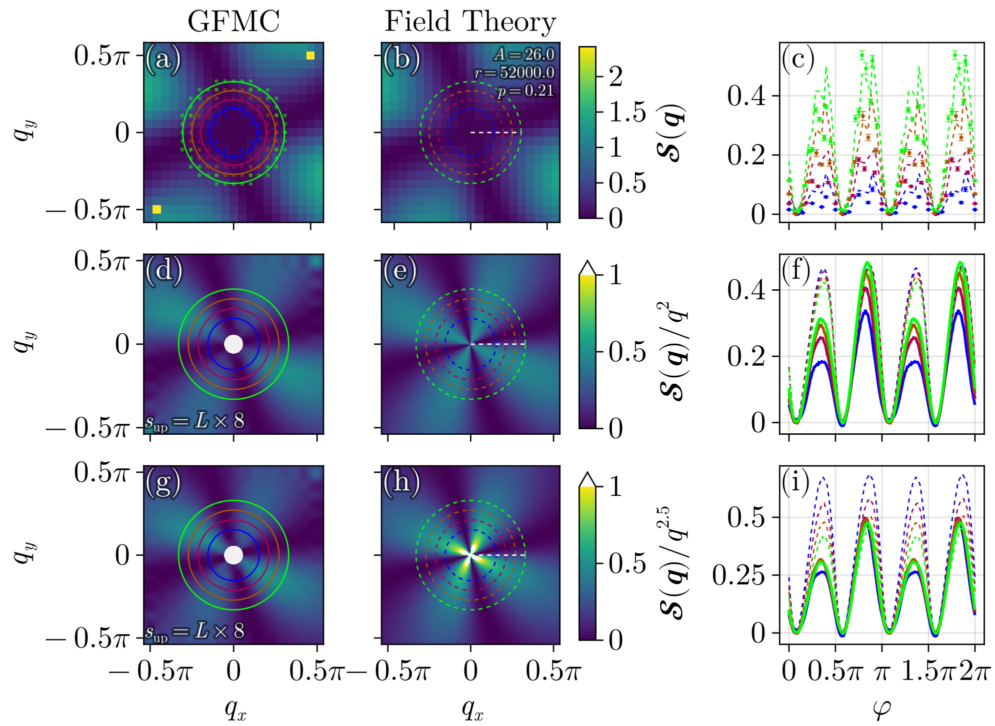

Figure 5: As in Fig. 2, but in the ordered phase for $\mu = 0.6J'$. Panels (g-i) show the collapse for a structure factor rescaled by a higher power $q^{2.5}$.

Response:

We agree with the Referee that the mere presence of nodal features with suppression at the center, while a necessary condition, is not sufficient to indicate the emergence of a fractonic quantum U(1) gauge theory. We argue, however, that the precise shape of the spin structure factor and the scaling of the suppression $\sim q^2$ are indeed indicative of a fractonic QSL with gapless photons and, consequently, disappear in an ordered phase. To demonstrate this, we repeat the analysis of the quadratic suppression shown in Figs. 2 and 3, but now within the *ordered* phase of the ground-state sector at $\mu = 0.6J'$. The corresponding plots are shown in Fig. 5, where Fig. 5(a)-(c) shows the unscaled data from GFMC and the field theory, while Fig. 5(d)-(f) shows the same data but rescaled by $\frac{1}{q^2}$. Importantly, the data collapse in Fig. 5(f) is largely destroyed compared to the spin-liquid phases in Fig. 2(i) and Fig. 3(i), indicating that the onset of magnetic long-range order is associated with a qualitative change of these nodal features. More precisely, the suppression in the ordered phase is much stronger than $\sim q^2$ in the spin-liquid phase and is better described by an empirical $q^{2.5}$ behavior; see Fig. 5(g)-(i).

This stronger suppression in the ordered phase can be explained by considering the limit $\mu \to -\infty$, which can be treated analytically. Here, we investigate this limit in the sector of the diagonal stripe state (ground-state sector), which we denote $|\psi_{\text{st}}\rangle$ (see Fig. 3(a), panel 6, in the manuscript for an illustration). Since the diagonal stripe state $|\psi_{\text{st}}\rangle$ is an eigenstate of $\mathcal{H}_1$ and $\mathcal{H}_3$, and since $\mathcal{H}_2$ is much smaller than $\mathcal{H}_1 + \mathcal{H}_3$, one may treat the effect of $\mathcal{H}_2$ on $|\psi_{\text{st}}\rangle$ perturbatively. In first

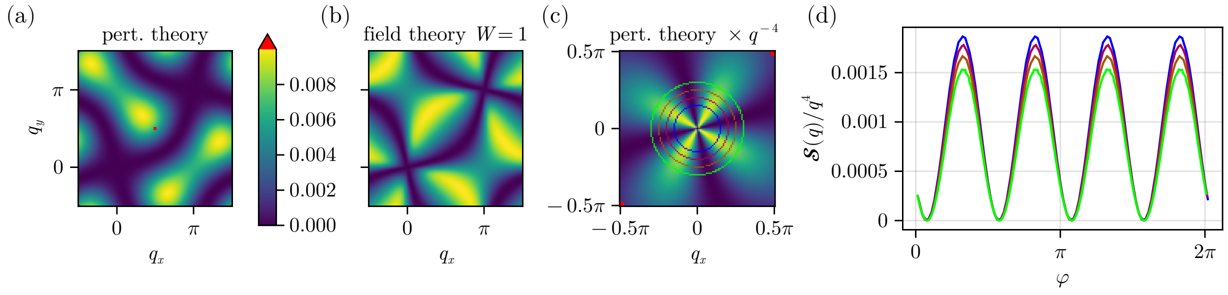

Figure 6: (a) Spin structure factor in the sector of the diagonal stripe state at $\mu \to -\infty$ computed in first order perturbation theory in $\mathcal{H}_2$. (b) Prediction of the spin structure factor of the fractonic U(1) field theory of Eq. (32) in the Supplementary Material at $W = 1$. (c) Same as (a) but rescaled by $\frac{1}{q^4}$ and plotted in a smaller part of the momentum space. (d) Spin structure factor in (c) plotted along the rings shown in (c).

order in $\mathcal{H}_2$, the perturbed state takes the form

$$|\psi^{(1)}\rangle = |\psi_{\rm st}\rangle - \frac{J'}{2\mu} \sum_{\square} \left(\mathcal{F}_{\square} + \mathcal{F}_{\square}^{\dagger} \right) |\psi_{\rm st}\rangle. \tag{2}$$

The spin structure factor $\mathcal{S}(\boldsymbol{q})$ of $|\psi^{(1)}\rangle$, shown in Fig. 6(a), features magnetic Bragg peaks at $\boldsymbol{q} = \pm(\pi/2, \pi/2)$ indicative of magnetic long-range order. Furthermore, the spin structure factor exhibits a diffuse signal from the application of fluctuator moves $\mathcal{F}_{\square}$ and $\mathcal{F}_{\square}^{\dagger}$ on $|\psi_{\rm st}\rangle$. This diffuse signal has the analytical form

$$\mathcal{S}_{\rm diff}(\boldsymbol{q}) \sim [\cos(q_x) - \cos(q_y) + 2\sin(q_x)\sin(q_y)]^2. \tag{3}$$

Interestingly, $\mathcal{S}_{\rm diff}(\boldsymbol{q})$ also has a characteristic structure of nodal features and suppressed pinch points. However, its precise shape is different from the spin structure factor of our fractonic U(1) gauge theory shown for $W = 1$ in Fig. 6(b) for comparison (see Eq. (32) in the Supplementary Material for the analytical expression). Specifically, as shown in Fig. 6(c) and (d), this first-order spin structure factor scales as q^4 for small momenta, explaining the observed suppression stronger than q^2 once magnetic long-range order sets in.

In summary, although nodal features and suppressed pinch points also occur in ordered phases, their shapes differ qualitatively from those in the spin liquid phase, exhibiting a stronger suppression at small momenta and thereby providing clear distinguishing features between the two phases.

Since we find this discussion, prompted by the Referee's insightful question, very important, we have added it in a new section in the Supplementary Material. In addition, we have revised the main text to avoid implying that the suppression of the spin structure factor by itself constitutes evidence for a fractonic quantum spin liquid.

Referee Comment

4. In Section V, the authors briefly mention the stability of the gapless phase against U(1) gauge fluctuations. It is well-established that in compact U(1) gauge theories in 2+1 dimensions, the monopoles can generate a mass gap for the photon, e.g., a confined phase. I would suggest the authors to carry out a careful scaling analysis in their Monte Carlo simulations to justify the gapless ground state.

Response:

It is indeed well known from the seminal work of Polyakov [Nuclear Physics B 120, 429 (1977)] that compact QED in 2+1 dimensions exhibits confinement due to monopole proliferation. In contrast, the fractonic rank-2 U(1) gauge theory considered here possesses substantially different properties, and we argue in our manuscript that the Polyakov confinement mechanism does not apply in this case. While the excellent agreement of the numerically obtained spin structure factor with field-theoretical predictions provides strong evidence for a genuinely deconfined fractonic quantum spin liquid, we agree with the Referee that additional analysis of the system's gaplessness would further strengthen this claim.

Although the GFMC method is inherently a ground-state technique and does not directly access the excitation spectrum, it nevertheless allows us to extract information through ground-state correlation functions. In particular, our GFMC results show that the spin-spin correlations exhibit a clear power-law decay, $\sim 1/|\boldsymbol{R}|^4$, persisting up to the largest accessible distances in the system. This behavior is illustrated in Fig. 7(b) for the ground-state sector at $\mu = 0.9J'$. Such algebraic decay is a hallmark of a gapless phase, in contrast to the exponential decay expected in a gapped system [Communications in Mathematical Physics 265, 781 (2006)]. Crucially, as we demonstrate analytically in a new section of the Supplementary Material, this $1/|\boldsymbol{R}|^4$ scaling is in exact agreement with the prediction of the rank-2 U(1) gauge theory with gapless photon modes.

Figure 7 also reveals additional structure in the real-space correlations. As shown in Fig. 7(a), the correlations are strongest within fan-like regions aligned with the Cartesian directions. These dominant contributions are highlighted as black dots in Fig. 7(b), while weaker correlations along other directions are indicated in gray. These results are now included in Fig. 4 of the manuscript, along with a brief discussion of the spatial structure of the correlations. A derivation of the $1/|\boldsymbol{R}|^4$ behavior is provided in the Supplementary Material.

Referee Comment

5. Even though it is a pure theoretical paper, it will be much better to discuss how to realize this interesting model in realistic systems? Such as Rydberg atoms?

Response:

We thank the Referee for raising this important question. While the original manuscript already contained a brief discussion of possible experimental realizations, we agree that a more detailed explanation is warranted and have expanded the discussion section accordingly in the revised version.

Overall, we consider a realization of our model in Rydberg atom arrays to be significantly more promising than in magnetic materials, although we emphasize that such an implementation remains experimentally challenging. On the positive side, the simple square-lattice geometry of

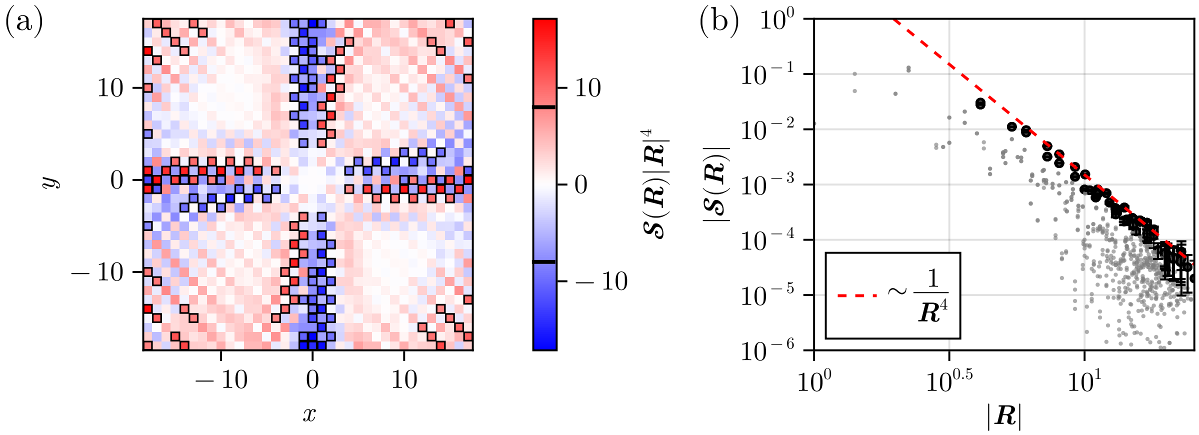

Figure 7: (a) Spin-spin correlation function $\mathcal{S}(\boldsymbol{R}) = \frac{1}{L^2}\sum_i \langle S^z(\boldsymbol{r}_i) S^z(\boldsymbol{R} - \boldsymbol{r}_i)\rangle$ rescaled by distance as $|\boldsymbol{R}|^4$ for the diagonal stripe sector at $\mu = 0.9J'$. (b) Decay of the dominant correlations (black points) along selected directions highlighted in (a), in comparison to the asymptotic power law $\sim 1/|\boldsymbol{R}|^4$, expected for a gapless fracton spin liquid in 2D. Gray dots indicate correlations along other directions.

the spiderweb model is well suited to current Rydberg platform capabilities. Moreover, a key simplifying aspect is that only the classical Ising part $\mathcal{H}_1$ needs to be engineered directly. Once the higher-rank Gauss law enforced by $\mathcal{H}_1$ is realized, weak quantum dynamics – e.g., via laser-induced local S^x terms – will generically generate the fluctuating terms contained in $\mathcal{H}_2$ perturbatively. We therefore discuss here possible routes to implement $\mathcal{H}_1$ (see also the revised discussion section for further details).

A central challenge is that standard Rydberg platforms naturally realize van-der-Waals interactions with a fixed spatial dependence $\sim 1/r^6$, implying that the relative interaction strengths between first-, second-, third-, and further neighbors are not independently tunable. Consequently, realizing $\mathcal{H}_1$ with independently adjustable coupling ratios requires more advanced techniques. Several recent proposals address precisely this limitation. For example, Floquet engineering schemes based on site-resolved periodic modulations of Rydberg states (PRL 135, 253001 (2025)) show that Hamiltonians with effectively programmable, distance-dependent spin–spin couplings can be realized. Similarly, multicolor Rydberg dressing protocols (PRR 4, L032046 (2022)) exploit multiple dressing lasers to generate several interaction channels with independently tunable strengths, thereby allowing substantial flexibility in designing effective spin models with nontrivial coupling patterns. While these approaches do not yet provide completely arbitrary coupling graphs, they appear sufficiently versatile to approximate the set of coupling ratios required in our model.

A second ingredient is the realization of effective spin-1 degrees of freedom in Rydberg systems, which typically encode spin-1/2 variables. Several promising routes have also been proposed in this direction such as multi-level encodings using multiple Rydberg states (PRX Quantum 6, 020332 (2025), arXiv:2502.06465, arXiv:2407.17554). These approaches are, in principle, compatible with the interaction-engineering techniques discussed above, although combining them in a single experimental setup would require careful optimization.

In summary, while a direct realization of $\mathcal{H}_1$ with fully independent coupling constants remains experimentally demanding, existing proposals for programmable interactions and multi-level encoding in Rydberg platforms suggest that an approximate implementation of the spiderweb model is within reach of near-term developments.

We believe that our responses and the corresponding changes to the manuscript have adequately addressed all the Referees' concerns. We thank the Referees again for their constructive feedback, which has significantly improved the manuscript.

Sincerely,
Nils Niggemann, Meghadeepa Adhikary, Yannik Schaden-Thillmann, Johannes Reuther

Response to Referee Reports

Manuscript: *Gapless fracton quantum spin liquid and emergent photons in a 2D spin-1 model*
Authors: *Nils Niggemann, Meghadeepa Adhikary, Yannik Schaden-Thillmann, Johannes Reuther*
Date: June 11, 2026

Dear Editor,

We thank the referees for their assessment and their endorsement for publication of our manuscript. We have included our responses below.

Response to Reviewer #1

We thank Reviewer #1 for their positive evaluation and for recommending publication. We address their clarifications below.

Referee Comment

I thank the authors for their detailed and constructive response. In my view, they have adequately addressed my main concerns, in particular by clarifying the distinction between the spin-1 and spin-1/2 cases, sharpening the meaning of quantum spin liquid in their context. Although the microscopic model remains somewhat engineered and contrived, I find the physical mechanism interesting and the results potentially valuable for the broader study of fractonic gauge structures and quantum spin liquids. I therefore recommend publication.

We thank the Referee for their endorsement for publication in nature communications.

Response to Reviewer #2

Referee Comment

I have carefully read the revised manuscript. I was satisfied with most of the authors' job addressing the reviewers' concerns.
I have only one minor observation regarding the discussion of the $q^{2.5}$ versus q^2 scaling added in the SM. While the sinc interpolation creates visually pleasing continuous circular cuts, I think that Distinguishing $q^{2.5}$ from q^2 based purely on finite-size numerical data requires extreme caution.
However, I understand and accept the authors' underlying physical argument: the suppression of the nodal features in the ordered phase is somehow faster than q^2. Therefore, the physical picture sounds and I have no further questions.
I would like to recommend the manuscript for publication.

We thank the Referee for their endorsement for publication in nature communications. We agree with their assessment regarding the difficulties of extracting the precise scaling relation and have

added a clarification emphasizing the more qualitative nature of this argument to our discussion in the supplementary material.

Sincerely,

Nils Niggemann, Meghadeepa Adhikary, Yannik Schaden-Thillmann, Johannes Reuther
